# Identification of a stromal immunosuppressive barrier orchestrated by SPP1^+^/C1QC^+^ macrophages and CD8^+^ exhausted T cells driving gastric cancer immunotherapy resistance

**DOI:** 10.3389/fimmu.2025.1618591

**Published:** 2025-07-16

**Authors:** Guichuang Ma, Xiaohan Liu, Qinrui Jiang, Shaowei Li, Qijing Wu, Bishan Liang, Fei Sun, Chunhui Gu, Wangjun Liao, Zhihua Zhang, Min Shi, Qiong Huang

**Affiliations:** ^1^ Department of Oncology, Nanfang Hospital, Southern Medical University, Guangzhou, Guangdong, China; ^2^ Cancer Center, The Sixth Affiliated Hospital, School of Medicine, South China University of Technology, Foshan, China

**Keywords:** gastric cancer, immune microenvironment, immunotherapy resistance, macrophages, exhausted CD8 + T cells, cellular interaction network

## Abstract

**Purpose:**

The heterogeneity of immune cells is a critical manifestation of gastric cancer (GC) heterogeneity and significantly contributes to immune therapy resistance. Although previous studies have focused on the roles of specific myeloid cells and exhausted CD8^+^ T cells in immune resistance, the immune cell interaction network and its spatiotemporal distribution in GC immune resistance remain underexplored.

**Methods:**

This study integrated multiple GC single-cell RNA sequencing, spatial transcriptomics, bulk-RNA sequencing, and single-cell immunotherapy datasets of our cohort (NFHGC Cohort). Methods such as single-cell subpopulation identification, transcriptomic analysis, spatial colocalization, cell communication network analysis and tissue immunofluorescence of gastric cancer were employed to investigate immune cell interactions and their molecular mechanisms in immune resistance.

**Results:**

By leveraging a comprehensive approach that integrates single-cell RNA sequencing, spatial transcriptomics, and bulk RNA-seq profiles, we identified 20 immune subsets with potential prognostic and therapeutic implications. Our findings suggest a stromal immunosuppressive network orchestrated by Macro_SPP1/C1QC macrophages and CD8_Tex_C1 T cells, which may form a barrier impeding antitumor immunity. Macrophage-derived MIF signaling appears to drive immunosuppression via the MIF-CD74/CXCR4/CD44 axis. Based on these observations, we developed a preliminary TME classification system using a gene signature derived from barrier-associated immune cell markers and unsupervised clustering.

**Conclusions:**

Our study identified a potential stromal immunosuppressive barrier in gastric cancer, driven by Macro_SPP1/C1QC macrophages and CD8_Tex_C1 T cells, which may contribute to immune dysfunction and therapy resistance. Molecular subtyping based on this barrier’s presence could inform personalized immune therapy strategies.

## 1 Introduction

Over the past few years, immune checkpoint inhibitors (ICIs) have achieved remarkable advancements in the treatment of gastric cancer (GC) (1, 2). However, the substantial spatiotemporal heterogeneity of gastric cancer (GC) often leads to a contradictory clinical outcome, where a high objective response rate (ORR) does not translate into prolonged overall survival (OS). Nearly 50% of patients develop primary or acquired resistance (3, 4).

TIME (Tumor Immune Microenvironment) is a multifaceted ecosystem, where immune cells assume both “anti-tumor” and “pro-tumor” roles through intricate intercellular interactions (5). In GC immunosuppressive cells (5), such as myeloid-derived suppressor cells (MDSCs) (6), regulatory T cells (Tregs), and exhausted T cells, facilitate immune resistance through the secretion of inhibitory factors (7, 8) and the expression of immune checkpoint molecules. Tumor cells, along with microenvironmental factors, collectively facilitate the accumulation of these immunosuppressive cells within the GC TIME. In addition, a study has shown that the remodeling of the immune niche, exemplified by the formation of fibroblast activation protein–positive cancer-associated fibroblasts (CAFs) and exhausted T cells, or SPP1^+^ TAMs (SPP1^+^ Tumor-Associated Macrophages) and THBS2^+^ CAFs, also contributes to immune resistance (9).

Given the critical role of TIME in immune resistance, our team previously developed the TMEscore (10, 11) evaluation system, successfully predicting the efficacy of GC immunotherapy by integrating immune cell infiltration characteristics and validating it through a Phase I clinical trial. However, bulk-RNA data as published in this previous study alone are insufficient to enable a deeper exploration of immune cell interactions and niche dynamics. In this study, we integrate multiple single-cell RNA sequencing (scRNA-seq), spatial transcriptomics (ST) and bulk-RNA sequencing datasets, to systematically map the heterogeneity of immune cells in GC. We identify an immunosuppressive barrier composed of three types of dysfunctional immune cell cluster (Macro_SPP1, Macro_C1QC and CD8_Tex_C1). Based on this discovery, we propose a barrier-associated immune classification for GC. Our results might offer a foundation for delving deeper into the immune resistance mechanisms in gastric cancer (GC) and for devising more impactful therapeutic strategies.

## 2 Methods

### 2.1 Acquisition of data

We sourced raw single-cell RNA sequencing data for gastric cancer cohorts from the GEO database (accession number GSE183904, including 29 samples of tumor tissue), bulk transcriptomic data (TCGA-STAD, including 323 tumor samples) along with clinical and survival information for stomach adenocarcinoma from the UCSC Xena database, and spatial transcriptomic data from the GEO database (GSE251950, including 3 tumor samples). Additionally, we retrieved melanoma patient data treated with immune checkpoint blockade from GEO (GSE100797, including 25 samples) and the KIM cohort dataset (12) (PRJEB25780, including 45 samples) from the TIDE website. All mRNA expression values were formatted in TPM, and analyses were conducted using R (version 4.2.0).

### 2.2 Patients and samples

We collected single-cell RNA sequencing (scRNA-seq) data from fresh tumor samples of 8 gastric cancer patients prior to immune checkpoint blockade (ICB) treatment at Nanfang Hospital (Guangzhou, China). Among the eight patients included in the analysis, F116 and F171 were identified as having progressive disease (PD), F153, F154, and F159 were identified as having stable disease (SD), and F128, F160, and F172 were identified as having partial response (PR). Here, PD and SD patients were considered non-responders to immunotherapy, while PR patients were considered responders. The determination of PD, SD, and PR was based on the Response Evaluation Criteria in Solid Tumors (RECIST 1.1) criteria (13). Written consent was obtained from all participants, and detailed clinical information is documented in **Supplementary Table S1**. Gastric cancer tissues used for immunofluorescence experiments were obtained from tumor biopsy samples of four patients with progressive disease (PD) and four patients with partial response (PR) who had received immunotherapy.

### 2.3 Single-cell data quality control and preprocessing

The CellRanger pipeline (version 4.0.0) developed by 10× Genomics was employed to process the raw sequencing data. This included alignment, quantification, basic filtering, and quality control to generate the initial gene expression matrix based on the human reference genome GRCh38. Subsequently, the R package Seurat (version 4.4.0) was utilized for downstream quality control and analysis. Datasets were preprocessed individually for each sample and then combined per donor to facilitate further analysis. During quality control, cells with a library size ≤2,000 UMIs or a mitochondrial transcript ratio ≥5% were excluded. Genes detected in fewer than 3 cells were marked as undetected. We merged the individual Seurat objects from each patient into a larger Seurat object and performed normalization, scaling, and preprocessing for PCA analysis. To assess batch effects, we first visualized data using a DimPlot. To correct for inter-sample variation, we applied the RunHarmony function from the R package Harmony.

The number of principal components (PCs) was determined by the JackStraw procedure using the JackStraw and ScoreJackStraw functions. Cell clustering was conducted with the FindNeighbors and FindClusters functions from Seurat, setting the resolution at 0.6. In the dataset GSE183904, a total of 115,134 cells were retained for further analysis, while the NFHGC cohort dataset retained 11,396 cells for further analysis.

### 2.4 Cell type annotation

Initial cell type annotations were performed using markers collected from published studies (14). For T cells,”*CD3D*”and”*CD8A*”were used for CD8^+^T cells, while “*CD4*” was used for CD4^+^T cells. For B cells, “*CD19*”, “*CD79A*”, “*IGHG1*”, and “*MZB1*” were used. Myeloid cells were distinguished using “*CD68*” and “*CD14*”; stromal cells were marked by “*PECAM1*”, “*VWF*”, “*COL1A1*”, and “*ACTA2*”; epithelial cells were marked by “*EPCAM*” and “*KRT8*”. To refine cell type annotations, the FindAllMarkers function was used to identify gene markers for each cell subtype, with criteria of adjusted P value ≤0.05 and average log fold change (avgLogFC) ≥0.15. The top 30 genes for each cell type were selected as representative markers, and the marker genes for the cell subpopulations are listed in **Supplementary Tables 2, 3**.

### 2.5 Spatial data processing and cell type deconvolution

Public spatial transcriptome datasets (GSE251950) were downloaded from GEO. For analysis, spots with fewer than 200 detected genes were filtered out, and genes with fewer than 10 read counts or expressed in fewer than 3 spots were removed. PCA was applied to reduce dimensionality on the log-transformed gene-barcode matrices of the top variable genes. Clustering was performed with a resolution of 0.8 to generate the final spot cluster results (15, 16).

Spatial deconvolution was performed using the RCTD algorithm from the R package spacexr-2.0.0 (17). For each spatial slice, matrices of raw counts and spatial coordinates were used to construct the Spatial RNA object. Integrated fibroblast cell raw counts and annotations served as the scRNA-seq reference with default parameters. The reference and Spatial RNA objects were then processed through the RCTD main function in full mode. For a certain cell type of each spot, the top score among cell types extracted from RCTD will be defined as the cell type. The AddModuleScore function was adapted to further identify the abundance and location of each detailed immune cell subtypes, using gene sets extracted from single-cell data as described above.

### 2.6 Spatial cell community analysis

The MISTy algorithm in the mistyR package (version 1.2.1) was utilized to evaluate the significance of the abundance of each primary cell type relative to other major cell types. Geneset scores from the AddModuleScore function and spatial coordinates were input to estimate cell-type-specific relationships. The Plot_interaction_communities function was used to visualize the final outputs with a cutoff value of 0.2.

### 2.7 Single-cell RNA CellChat analysis of cell–cell communication

To analyze single-cell level cell–cell communication, the R package CellChat (version 1.6.1) was used. Ligand-receptor interactions were constructed using the human dataset from CellChatDB. Communication probabilities were calculated with the computeCommunProb function, and interactions involving fewer than 5 cells were excluded. Aggregated cell communication patterns were then used to select signals between immune cells and other tumor microenvironment (TME) cells based on spatial co-location.

### 2.8 Single-cell functional score

We separately collected the functional gene sets of B cells, T cells, and macrophages from previously published manuscripts (18–20), and used the AUCell method to score them. For each gene set, after calculating, the scores were integrated into the metadata of the cell Seurat. The functional gene sets are listed in **Supplementary Table 4**.

### 2.9 Single-cell RNA cell trajectory analysis

The Monocle2 package (version 2.18.0) was used to infer cell trajectories for CD8^+^ T cells. “Dispersion” genes were identified using the estimate Size Factors and estimate Dispersions functions and used to order cells. The trajectory was constructed using the DDRTree method for dimensionality reduction and plotted in three-dimensional space.

### 2.10 Network motif analysis

The CellChat interaction strength matrix among immune cells was extracted as an adjacency matrix, excluding weak interactions with weights less than 0.0001. The root node was determined as the node with the highest weighted outdegree. To identify immune cell interaction patterns, 2-node, 3-node, and 4-node motifs were sequentially detected using the “IGLADFindSubisomorphisms” function from the IgraphM package in Mathematica. The “IGRewire” function was used to detect sub-motifs by generating 50,000 degree-preserving random networks. The sum strength weight was calculated to determine the interaction significance of each motif.

### 2.11 Bulk transcriptomes analysis

To compare cell abundance between responder (R) and non-responder (NR) patients to ICB therapy, the ssGSEA method in the GSVA package was used to calculate cell type scores. The TimiGP package was used to distinguish ICB (Immune Checkpoint Blockade) response status. Gene sets were selected as described above, and the TimiPreProcess function was used to prepare the data. The TimiCellPair, TimiBG, and TimiCellNetwork functions were used to estimate the FS score contributing to ICB treatment. For sub-clusteRing of immune conditions among gastric cancer patients, immune cell scores, epithelial cell scores, and stromal cell scores were initially estimated and then further clustered using the k-means method from the “me_cluster” function in the IOBR 2.0 package.

### 2.12 Identification of barrier-associated immune classification *via* bulk transcriptomes analysis

The identification of the barrier-associated immune classification was calculated with the help of the IOBR package. We then used the extracted single-cell gene set to perform ssGSEA (single-sample gene set enrichment analysis) scoring for each TCGA sample. After that, we use the tme-cluster function for clustering, and the method is set as “ward.D2”. In order to obtain the best clustering effect, we set the minimum number of clusters to 2 and the maximum to 10. We use the function sig_heatmap for visualization.

### 2.13 Survival analysis

Clinical survival information for TCGA-STAD was obtained from the UCSC Xena database. To assess the correlation between immune cells and patient survival, gene set expression values were extracted from the single-cell dataset, and the top 30 genes were used to calculate immune cell scores. The optimal cut-off values for immune cell score levels, based on ssGSEA scores, were determined using the Surv_cutpoint function from the R survminer package with the Kaplan-Meier method. Patients were then divided into high and low infiltration groups, and Kaplan–Meier survival curves were fitted and visualized using the survfit and ggsurvplot functions to assess the impact of infiltration levels on survival outcomes.

### 2.14 Development of nomogram for ICB response

In the bulk ICB treatment cohort, the cell abundance score from ssGSEA were incorporated into the analyses for the response of ICB treatment. The relationship between immune cell infiltration and treatment response was assessed using the lrm fitting function, with variables having *P* < 0.05 selected for subsequent analyses.

### 2.15 TF target network prediction

The input data consisted of immune cell Seurat objects, and TF-gene relationships were extracted using the the get_collectri function. TF enrichment scores were inferred using the run_ulm function (21).

### 2.16 Drug prediction

To predict relevant drugs targeting specific cells, the drug2cell module was implemented using single-cell data in the Python environment (22). Input data was the public gastric cancer dataset, and the celltype label are observations to calculate potential drugs.

### 2.17 Immunofluorescence

Multiple immunofluorescence experiments were performed according to the manufacturer’s instructions using a Quadruple Fluorescence IHC Mouse/Rabbit Kit (Immunoway, RS0037). Paraffin-embedded patient tissues were sectioned at a thickness of 4 μm, dewaxed, and subjected to antigen retrieval in EDTA buffer (pH 8.0) for 15 minutes using a microwave oven. All sections were subjected to four rounds of staining. The primary antibodies used were as follows: anti-CD8A (1:200, CST); anti-CD206 (1:200, Proteintech); anti-SPP1 (1:200, Proteintech); anti-C1QBP (1:200, Proteintech); anti-PD1 (1:200, Proteintech); anti-TIM3 (1:200, Proteintech); and anti-MIF (1:200, Proteintech). Finally, images were captured using a fluorescence microscope and a confocal laser microscope (Nikon). Fluorescence intensity was quantified using ImageJ software.

### 2.18 Statistical analyses

Comparisons between two groups were made using Student’s t-test, whereas one-way ANOVA was applied for comparisons involving more than two groups. Survival rates were assessed using the Kaplan–Meier method. Statistical significance was set at *P* < 0.05.

## 3 Results

### 3.1 Single-cell transcriptomics reveal the heterogeneity of immune cells in gastric cancer

**Figure 1:**
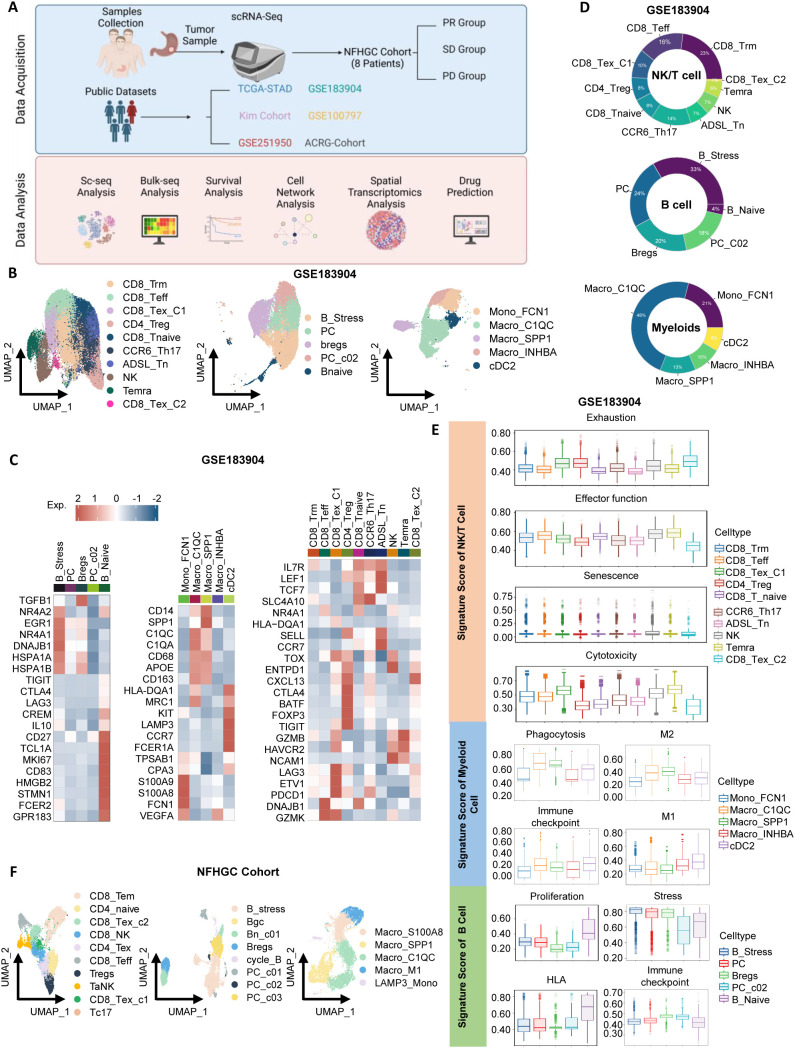
Identification of immune cells in gastric cancer. **(A)** Schematic overview of the study design. **(B)** UMAP visualization of subclusters of NK/T cells, B cells, and myeloid cells from the GSE183904 dataset. **(C)** Heatmap highlighting gene markers of immune cell subclusters. **(D)** Donut chart depicting the proportions of immune cell subclusters. **(E)** Violin plots displaying signature scores of immune cell functions. **(F)** UMAP visualization of subclusters of NK/T cells, B cells, and myeloid cells from the Nanfang Hospital Gastric Cancer Immunotherapy Cohort (NFHGC Cohort, n=8 patients).

To thoroughly investigate the heterogeneity of the immune microenvironment in gastric cancer, we integrated multiple datasets for comprehensive analysis (**Figure 1A**). First, for the gastric cancer samples in the GSE183904 dataset, we identified three major immune cell types: NK/T cells, myeloid cells, and B cells (**Supplementary Figures S1A, B**). By referring to cell markers reported in the literature, we further classified these cell types into multiple subpopulations (**Figures 1B, C**) and displayed the proportions of each subpopulation using pie charts (**Figure 1D**). Among T cells, we identified 10 subpopulations (**Figures 1B, C**), including NK cells, CD4^+^T cells (Treg, CCR6_Th17, and ADSL_Tn) (23), and CD8^+^T cells (naive CD8^+^T cells [CD8_Tnaive], tissue-resident memory CD8^+^T cells [CD8_Trm], terminally differentiated memory CD8^+^T cells [Temra], effector CD8^+^T cells [CD8_Teff], and two exhausted subpopulations [CD8_Tex_C1 and CD8_Tex_C2]). B cells were divided into five groups (**Figures 1B, C**): naive B cells (Bnaive), stress-related B cells (B_Stress), two types of plasma cells (PC and PC_C02), and regulatory B cells (Breg). Notably, B_Stress cells highly expressed stress-related genes such as NR4A2 and EGR, while Bregs exhibited high expression of immune checkpoint molecules. Myeloid cell subpopulations included Mono_FCN1, Macro_C1QC, Macro_SPP1, Macro_INHBA, and cDC2 (**Figures 1B, C**).

Next, using ssGSEA analysis, we summarized the functional characteristics of each immune subpopulation (**Figure 1E**), with most results consistent with previous literature, thereby validating the reliability of our clustering. Specifically, among T cells, CD8_Tex_C1, CD4_Treg, and CD8_Tex_C2 exhibited high exhaustion scores and the lowest levels of cytotoxicity and activation; in contrast, CD8_Teff cells showed the strongest adhesion capacity. In B cells, Bnaive cells demonstrated the highest proliferation and HLA antigen-presenting ability, while B_Stress, PC, and Breg cells exhibited high stress scores, suggesting their potential involvement in complex stress responses within the gastric cancer immune microenvironment. Among myeloid cells, Macro_C1QC and Macro_SPP1 displayed strong M2 macrophage features and expressed numerous immune checkpoint molecules, indicative of immunosuppressive properties. Interestingly, these two cell types also exhibited robust phagocytic functions.

In the Nanfang Hospital gastric cancer immunotherapy cohort (NFHGC cohort), we further identified multiple functional subpopulations of NK/T cells, B cells, and myeloid cells (**Figure 1F**) and found that their immune phenotypes and functional characteristics were highly consistent with those in the GSE183904 dataset, presenting similar patterns (**Supplementary Figures S1C, D, H; S1E–G**).

### 3.2 Immune cell subpopulations associated with the efficacy of immune therapy in gastric cancer

**Figure 2:**
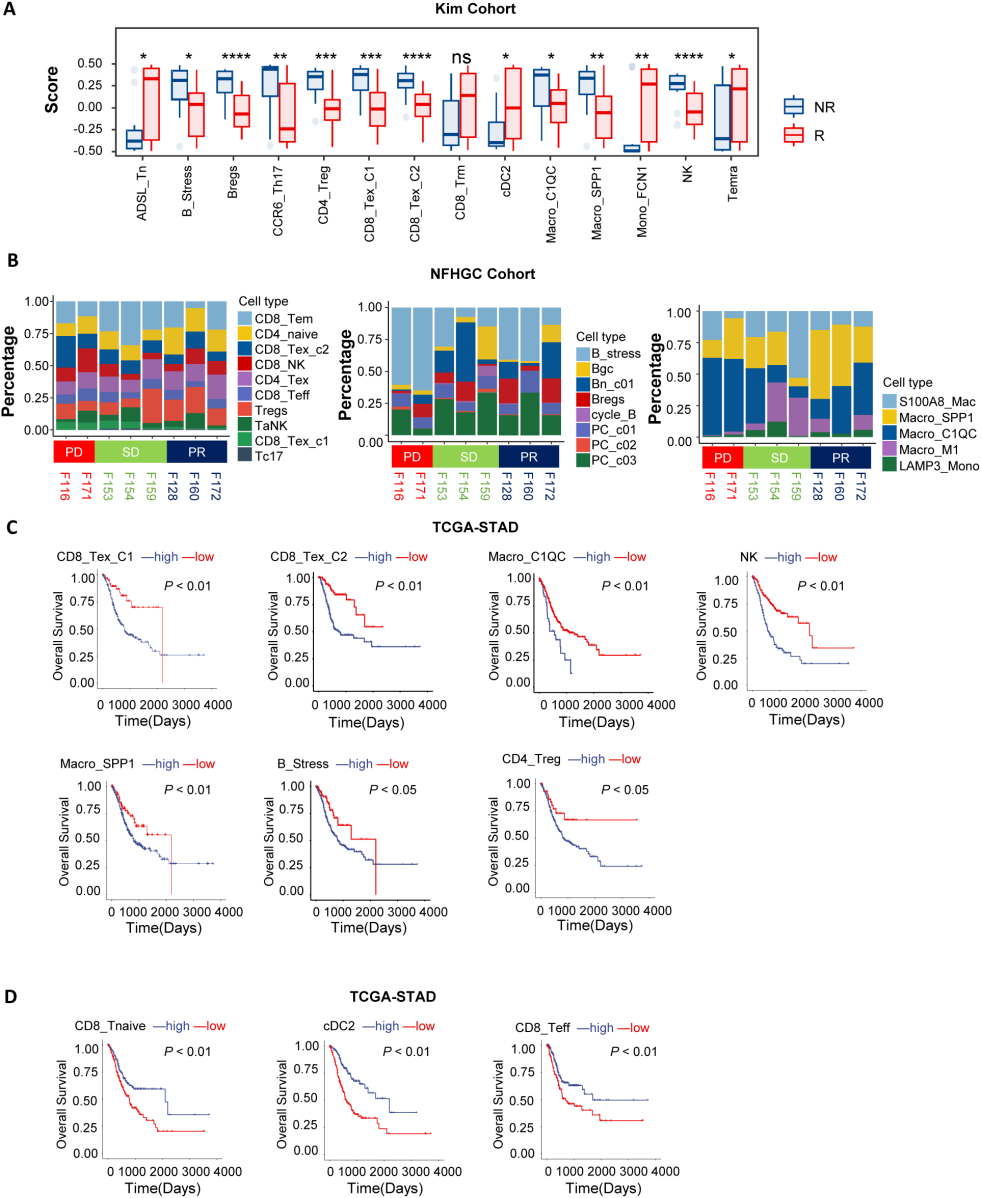
Association of immune cells with patient clinical outcomes. **(A)** Box plots showing differences in immune cell deconvolution scores between immunotherapy responders (R) and non-responders (NR) in the KIM cohort. Asterisks (*) indicate statistical significance (*P* < 0.05), whereas “ns” denotes non-significance. **(B)** The bar chart illustrates differences in immune cell proportions among patients with distinct immunotherapy outcomes (PD, SD, PR) in the NFHGC Cohort. **(C, D)** Survival analysis in TCGA-STAD based on immune cell marker gene deconvolution scores. Patients were stratified into high- and low-score groups for survival analysis. **(C)** Immune cell types associated with poor prognosis. **(D)** Immune cell types associated with favorable prognosis. **(A)** *P* value < 0.05 was considered statistically significant. **P < 0.01, ***P < 0.001, and ****P < 0.0001.

To investigate the association between immune cell subpopulations and the efficacy of immune therapy in gastric cancer, we conducted deconvolution-based cell enrichment scoring in the bulk-transcriptome cohort receiving immune therapy (KIM cohort). The analysis revealed significant enrichment of ADSL_Tn, CD8_Trm, cDC2, Mono_FCN1, and Temra cell subpopulations in the immune therapy responder group (R). In contrast, in the non-responder group (NR), immune-suppressive subpopulations such as B_Stress, Bregs, CD4_Treg, CD8_Tex_C1, CD8_Tex_C2, Macro_C1QC, and Macro_SPP1 were more abundant (**Figure 2A**). Consistent results were also noted in the melanoma cohort (**Supplementary Figure S2A**). Additionally, in the NFHGC single-cell cohort, the proportions of CD8_Tex_C1, CD8_Tex_C2, B_Stress, and Macro_C1QC subpopulations were higher in patients from the NR group, especially in those with PD-L1 expression (**Figure 2B**).

To further elucidate the prognostic value of these cell subpopulations in gastric cancer survival, we conducted survival analyses in the TCGA-STAD cohort. The results indicated that high enrichment of CD8_Tex_C1, CD8_Tex_C2, Macro_C1QC, Macro_SPP1, NK, CD4_Treg, and B_Stress subpopulations—which were more abundant in the NR group—was associated with poor prognosis (**Figure 2C**). In contrast, enrichment of CD8_Tnaive, CD8_Teff, and cDC2 cells was associated with better prognosis (**Figure 2D**).

In summary, we identified two distinct groups of immune cell subpopulations in the gastric cancer immune microenvironment that are associated with opposite outcomes in immune therapy response and clinical prognosis. Enrichment of CD8_Tex_C1, CD8_Tex_C2, Macro_C1QC, Macro_SPP1, NK, CD4_Treg, and B_Stress cells is associated with immune resistance and represents a ‘pro-tumor’ faction. In contrast, enrichment of CD8_Tnaive, CD8_Teff, and cDC2 cells is indicative of better treatment response and constitutes an ‘anti-tumor’ faction. The dynamic changes of these two groups of cells can serve as potential predictive biomarkers for the efficacy and prognosis of immune therapy.

### 3.3 Hierarchical interactions of immune cells in gastric cancer

Immune cells do not exist in isolation but form complex networks through interactions to regulate immune responses. Analysis of cell communication revealed intricate signaling interactions between immune cells of different clusters, with cellular crosstalk potentially driving both anti-tumor and pro-tumor effects simultaneously. Based on the upstream-downstream relationships in the communication strength matrix and immune cell functions, we defined three functional modules: pro-tumor (M1), anti-tumor (M2), and anti-promoting interaction (M3) modules (**Supplementary Figures S2A–C**).

**Figure 3:**
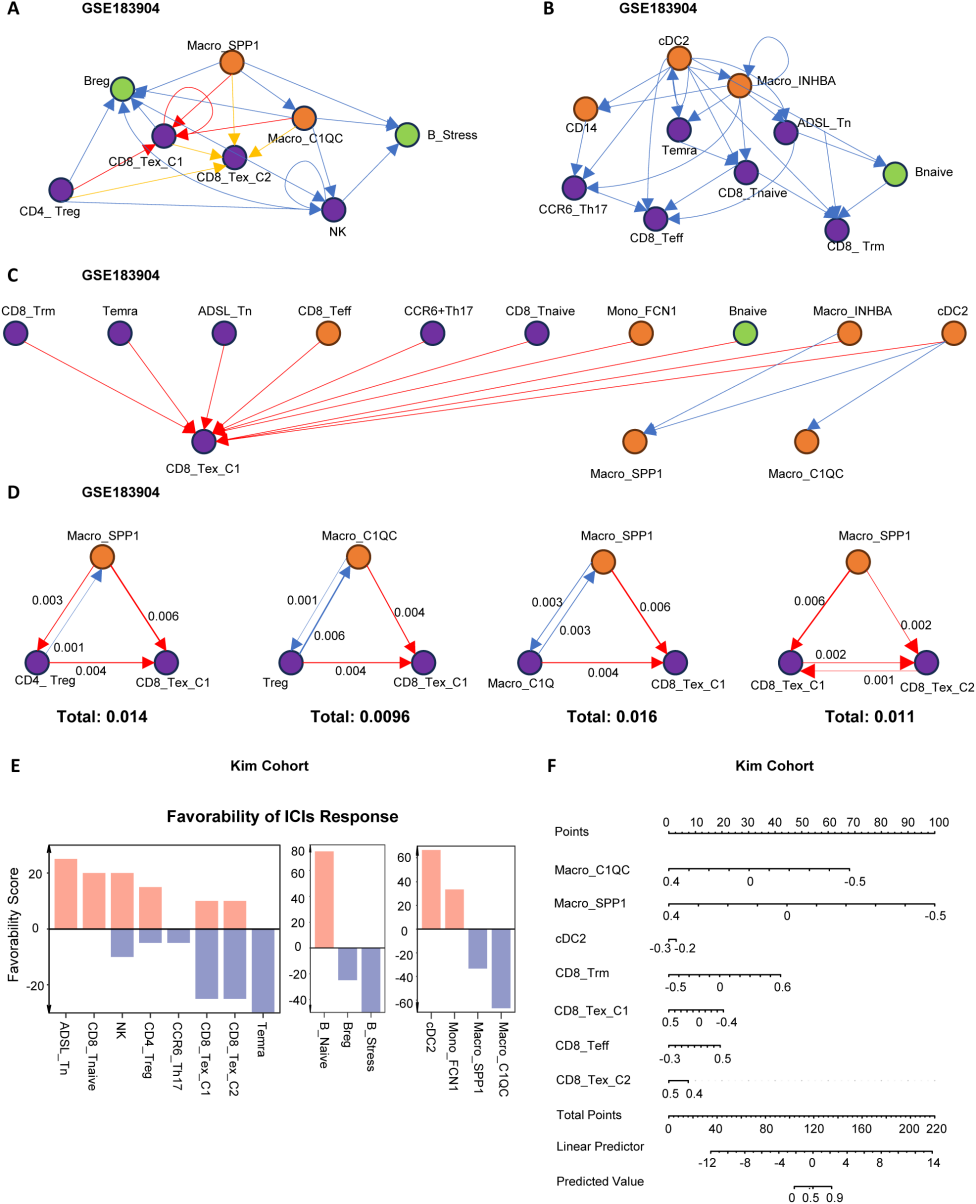
Network analysis of immune cell communication hierarchies. **(A–C)** Hierarchical network structures based on ligand-receptor communication strength among immune cells. The root node was defined as the node with the highest weighted outdegree. **(A)** Hierarchical structure of pro-tumoral immune cell modules. **(B)** Hierarchical structure of anti-tumoral immune cell modules. **(C)** Hierarchical structure of coordinated immune cell modules. Module classification was determined based on immune cell function and their impact on clinical outcomes. **(D)** The top four scoring three-cell motif subgraphs. Arrow thickness indicates the strength of cell communication, and the total score represents the sum of communication strength scores within the three-cell motifs. **(E)** Bar plot displaying Favorability Scores of cell subtypes for ICIs treatment response in the KIM cohort. Higher Favorability Scores indicate better immunotherapy response. **(F)** Nomogram illustrating the predictive ability of immune cells for immunotherapy response in the KIM cohort. Higher Predicted Values indicate better immunotherapy response.

To further elucidate the interaction network and hierarchical structure of immune cells, we used the igraphM function in Mathematica, extracting communication strengths from CellChat as an adjacency matrix to construct a directed cellular network (24). Within the defined M1 to M3 modules, we selected the cells with the strongest signaling output as root nodes (**Figures 3A–C**). In the anti-tumor M2 module, cDC2 was positioned at the most upstream level, capable of activating Macro_INHBA, Mono_FCN1, and various T cells, while CD8_Teff and Trm were downstream recipients of regulatory signals (**Figure 3B**). This process aligns with the immune cycle of DC activation of T cells exerting anti-tumor effects. In the pro-tumor M1 module, SPP1/C1QC^+^ macrophages were at the top, primarily mediating varying degrees of CD8^+^T cell exhaustion, highlighting the central role of macrophage-T cell interactions in immune dysfunction (**Figure 3A**). In the anti-promoting interaction M3 module, cDC2 was again at the most upstream level, interacting with CD8_Tex_C1 cells and SPP1/C1QC^+^macrophages. This is associated with excessive antigen presentation and stimulation-mediated functional exhaustion, further revealing the mutual “Tug-of-War” between anti-tumor and pro-tumor immune functions (**Figure 3C**).

To further clarify the cellular relationships in the pro-tumor (M1) module contributing to immune therapy resistance, we identified the most common immune dysfunction interaction motifs, finding that motifs composed of three cells were dominant (**Supplementary Figures S2E, F**). By quantifying the strength of these motifs, we found that Macro_SPP1 and Macro_C1QC, as upstream cells in immune dysfunction, directly transmitted regulatory signals to Treg, CD8_Tex_C1, and CD8_Tex_C2. CD8_Tex_C2, as the most downstream and terminally exhausted cell, was also regulated by Treg and CD8_Tex_C1 (**Figure 3D**).

Subsequently, we used the TimiGP (25) algorithm to quantify the “favorability score” (FS) of key cells in immune therapy (**Figure 3E**). The results showed that CD8_Tex_C1, CD8_Tex_C2, Bregs, B_stress, Macro_C1QC, and Macro_SPP1 had negative FS values, indicating that these cells might have detrimental effects on immune therapy. Further analyses using nomograms in the KIM cohort (**Figure 3F**) and the melanoma cohort (**Supplementary Figure S2G**) confirmed the significant role of the “Macro_SPP1-Macro_C1QC-CD8_Tex_C1” triad in immune resistance.

### 3.4 Spatial localization features of anti-tumor M2 and pro-tumor M1 module cells

**Figure 4:**
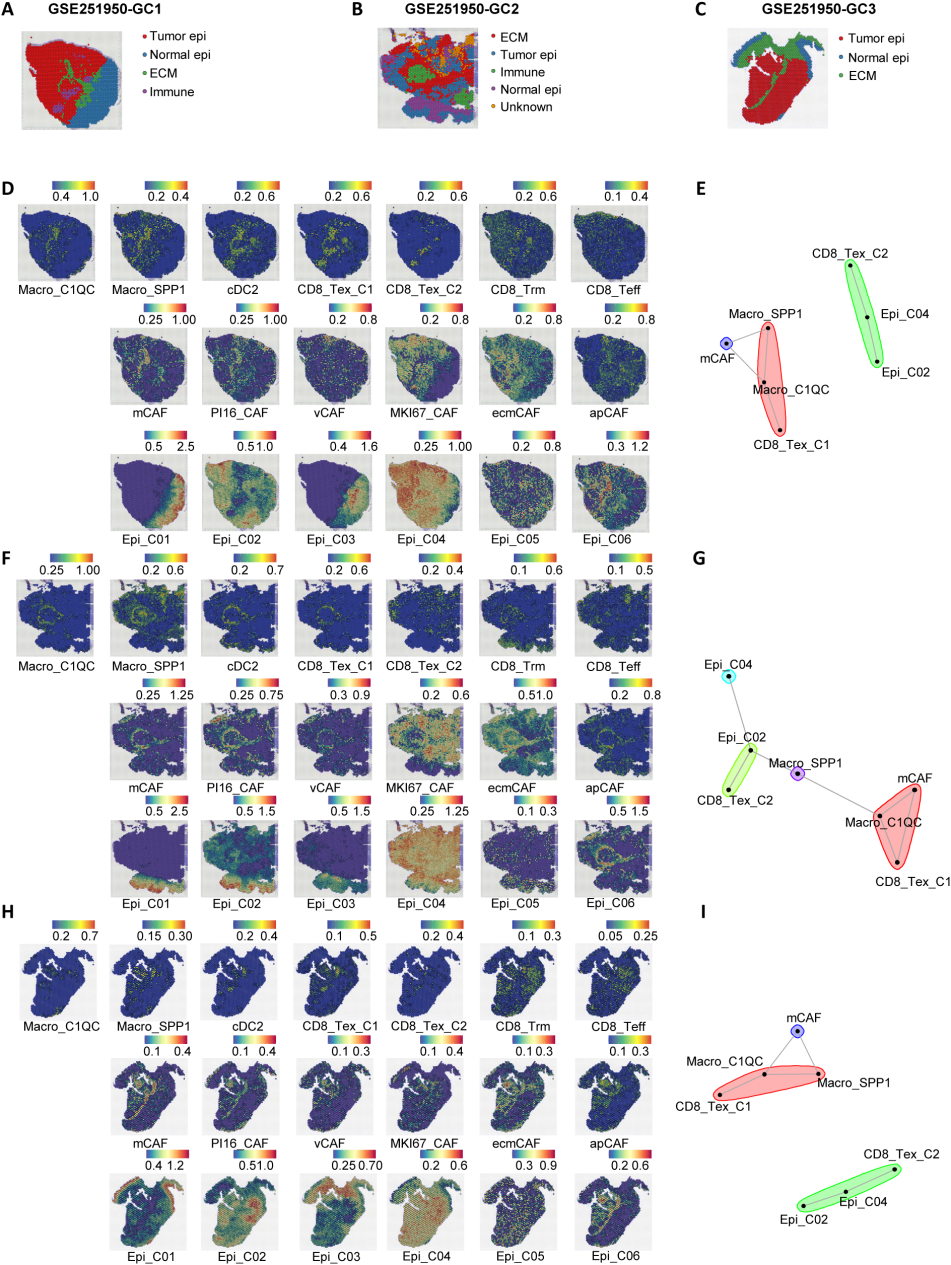
Spatial transcriptomics reveal a stromal immunosuppressive barrier. **(A–C)** Spatial architecture and relationships in three spatial transcriptomic sections (GC1, GC2, and GC3) from the dataset GSE251950. **(D)** Abundance of immune cells, epithelial cells, and CAFs based on signature scores in the spatial data of GC1. **(E)** Spatial cell network community plot of the GC1 section. For example, the green cluster represents continuous interactions among CD8_Tex_C2, Epi_C04, and Epi_C02. **(F)** Abundance of immune cells, epithelial cells, and CAFs based on signature scores in the spatial data of GC2. **(G)** Spatial cell network community plot of the GC2 section. **(H)** Abundance of immune cells, epithelial cells, and CAFs based on signature scores in the spatial data of GC3. **(I)** Spatial cell network community plot of the GC3 section.

To further investigate the spatial localization characteristics of anti-tumor M2 and pro-tumor M1 module cells, we selected three gastric cancer spatial transcriptome sections (GC1, GC2, and GC3) from the public dataset GSE251950 (26) (**Figures 4A–C**). Through unbiased clustering and spot feature analysis, the sections were divided into tumor cell areas, normal epithelial areas, stromal cell areas, and immune areas (**Figures 4A–C**), and the subpopulations of cells in each module were identified. The analysis revealed spatial heterogeneity of M1 and M2 module cells, with Macro_C1QC, Macro_SPP1, and CD8_Tex_C1 predominantly enriched in the stromal area, while CD8_Tex_C2 exhibited higher abundance in the tumor area. In contrast, cDC2, Trm, and Teff cells in the M2 module did not show distinct spatial distribution patterns, which may be related to their tissue-resident characteristics.

Beyond immune cell communication, tumor epithelial cells and fibroblasts are also crucial in interacting with immune cells. To further investigate the spatial interactions between M1 module immune dysfunction cells and other microenvironmental cells, we identified six epithelial cell subpopulations (27, 28) (Epi01-06, **Supplementary Figures S4A, C, S7A–D**) and six stromal cell subpopulations (apCAFs, ecmCAFs, mCAFs, MKI67_CAFs, PI16_CAFs, vCAFs, **Supplementary Figures S4B, D, S7A–D**) in the GSE183904 and NFHGC single-cell cohorts. In addition, we analyzed the expression differences of the *THY1* gene across various cell subpopulations in the GSE183904 dataset and the NFHGC cohort. Interestingly, *THY1*, traditionally recognized as a pan-T cell marker in mice and reported in the literature to encode CD90 and be expressed in fibroblasts (29–31), showed a distinct cell-type-specific expression pattern in both the GSE183904 and NFHGC datasets—being enriched in fibroblasts and cancer-associated fibroblasts (CAFs), but minimally expressed in T cell clusters (**Supplementary Figures S7E, F**). This context-dependent distribution suggests a stromal-associated role for *THY1* within the tumor microenvironment. Subsequently, we extracted the top 30 feature genes of these cells and used MISTy (32) (Multiview Intercellular Spatial modeling framework) to analyze the spatial proximity between immune, stromal, and epithelial cells (**Figures 4D, F, H**). The results showed high spatial proximity between Epi02 and Epi04 with CD8_Tex_C2, as well as significant spatial adjacency between Macro_SPP1, Macro_C1QC, CD8_Tex_C1, and mCAFs (**Supplementary Figures S4H–J**). Further cell community network analysis yielded similar findings (**Figures 3E, G, I**).

Additionally, single-cell communication analysis (**Supplementary Figure S4E**) revealed that the communication between mCAFs and Macro_SPP1, Macro_C1QC, CD8_Tex_C1, as well as between Epi_C04 and CD8_Tex_C2, was the most intense. Moreover, these communications were significantly upregulated in patients with poor immune therapy response (NR) (**Supplementary Figures S4F, G**).

In summary, we propose a stromal-localized immunosuppressive network coordinated by Macro_SPP1/C1QC macrophages and CD8_Tex_C1 exhausted T cells. This network forms a peripheral barrier that impedes antitumor immunity, primarily distributed outside the tumor core and infiltrating the stromal area dominated by mCAFs. Within the tumor core, which is mainly composed of Epi_C02 and Epi_C04 cells, CD8^+^ T cells are present. These CD8^+^ T cells likely migrate from the peripheral stromal area, where they are influenced by signals from Macro_SPP1/C1QC macrophages, leading to the attenuation of their antitumor functions. Upon entering the tumor core, antigens from Epi_C02 and Epi_C04 cells may further induce the transition of CD8_Tex_C1 cells to a terminally exhausted CD8_Tex_C2 state.

### 3.5 MIF signaling plays a critical regulatory role in the immunosuppressive network

**Figure 5:**
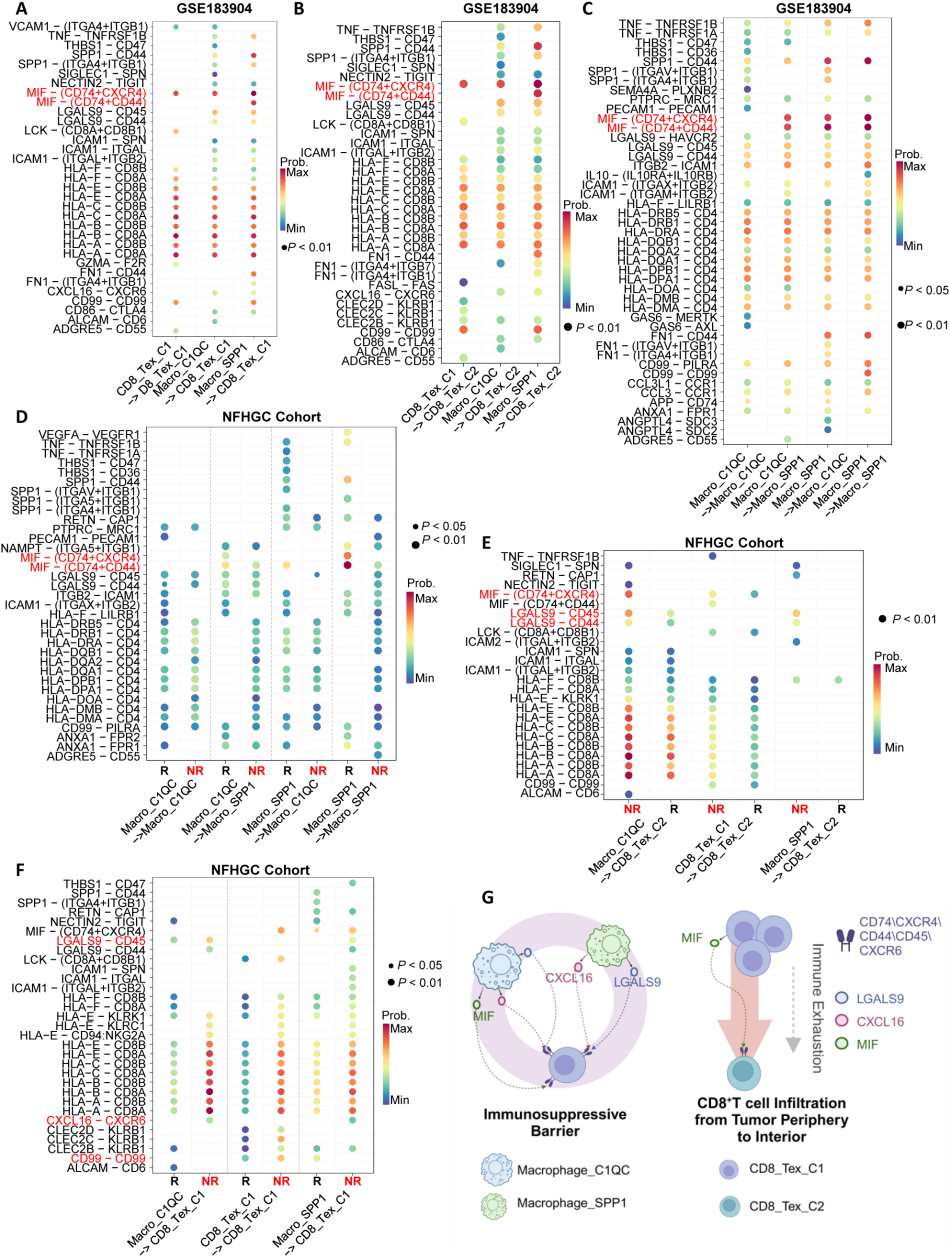
Identification of immune cell communication signals mediating ICIs non-response. **(A–C)** Ligand-receptor pair dot plots among immune cells in the GSE183904 dataset. The arrow (−>) indicates the direction of signaling from a sender cell subcluster to a receiver. **(D–F)** Ligand-receptor pair dot plots among immune cells in the NFHGC cohort. The arrow (−>) indicates the direction of signaling from a sender cell subcluster to a receiver. R denotes immunotherapy responders, and NR denotes non-responders. *P* < 0.05 indicates statistical significance. **(G)** Schematic illustration of communication mechanisms among immune cells in the immunosuppressive barrier. The barrier comprises Macrophage_SPP1, Macrophage_C1QC, and CD8_TEX_C1, while CD8_Tex_C2 is derived from CD8_Tex_C1 during infiltration into the tumor core, undergoing functional exhaustion and numerical decline. On the barrier, Macrophage_SPP1 and Macrophage_C1QC cells signal to CD8 cells via MIF, LGALS9 and CXCL16, mediating ICIs non-response in gastric cancer.

To elucidate the specific molecular mechanisms of cell-cell interactions within the immune interacting network, we first identified the major ligand-receptor communications in the GSE183904 cohort (**Figures 5A–C**). Subsequently, we compared the ligand-receptor communications between immune therapy responders (R) and non-responders (NR) in the NFHGC cohort (**Figures 5D–F**). The results showed that in the NR group, signaling from Macro_SPP1 to CD8_Tex_C1-including MIF-CD74/CXCR4/CD44, LGALS9-CD45, HLA-CD8, and CXCL16-CXCR6-was significantly enhanced. In contrast, the signal intensity changes between the two types of macrophages (Macro_SPP1 and Macro_C1QC) were not significant. Additionally, autocrine MIF and CD99 in CD8_Tex_C1 were significantly upregulated in the NR group. Further analysis revealed that, within the tumor, the LGALS9-CD44 signaling pathway from Macro_SPP1 macrophages to CD8_Tex_C2 T cells was upregulated in the non-responder (NR) group. Thus, within the immune interaction network, MIF, LGALS9, and CXCL16 signaling pathways appear to be the primary drivers promoting the functional decline of CD8_Tex_C1 cells. Furthermore, the transition of CD8_Tex_C1 cells to the CD8_Tex_C2 state, mediated by MIF and LGALS9 signaling, could potentially facilitate the emergence of immune resistance. (**Figure 5G**).

To elucidate the molecular mechanisms underlying the transition from CD8_Tex_C1 to CD8_Tex_C2, as well as the differences between these two cell populations, we conducted cell trajectory analysis. (**Supplementary Figure S5A**). The results indicated that both CD8_Tex_C1 and CD8_Tex_C2 originate from CD8_Tnaive, with a high degree of overlap in their differentiation trajectories. CD8_Tex_C2 tends to progress towards a terminal exhaustion state, consistent with the evolutionary trajectory of T cell exhaustion reported in the literature (33) (**Supplementary Figure S5E**). Subsequently, we comprehensively compared the differences in upstream transcription factors, immune checkpoint molecule expression, and KEGG pathway enrichment between the two cell populations (**Supplementary Figures S5B, C**, **Supplementary Table S4**). The transcription factors associated with CD8_Tex_C1 include TEAD1, KLF13, IRF6, NFKBIB, and RUNX1, while CD8_Tex_C2 is primarily regulated by E2F4, E2F1, and TFDP1. Regarding immune checkpoint molecules, CD8_Tex_C1 mainly expresses VSIR, TNFRSF9, and PDCD1, whereas CD8_Tex_C2 predominantly expresses TNFSF14, ICOS, and CTLA4. Functional pathway analysis showed that CD8_Tex_C1 is enriched in apoptosis and TNF signaling pathways, while CD8_Tex_C2 is enriched in multiple metabolism-related pathways, including glycolysis (**Supplementary Table S5**). These differences provide potential targets for precisely targeting different types of CD8^+^T cell exhaustion.

**Figure 6:**
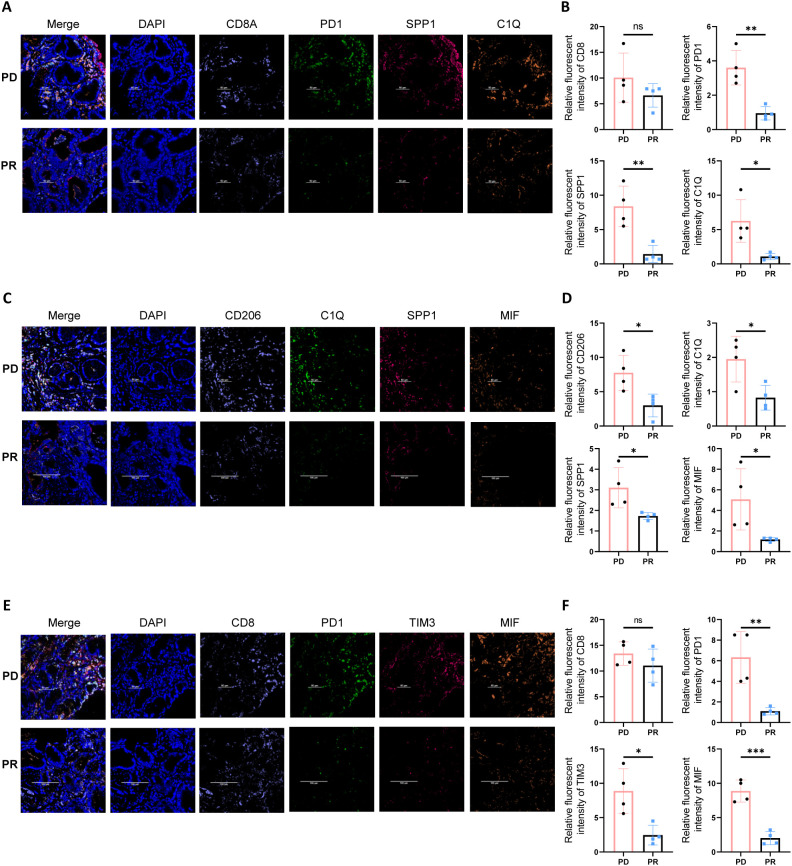
Multiplex immunofluorescence staining reveals an immune barrier structure in the tumor periphery of PD gastric cancer patients. **(A)** Multiplex immunofluorescence staining indicates that in the tumor periphery of PD gastric cancer patients, there is higher expression of PD1, SPP1, and C1Q. **(B)** Quantitative analysis of fluorescence intensity for CD8, PD1, SPP1, and C1Q. Asterisks (*) indicate statistical significance, whereas “ns” denotes non-significance. **(C)** In the tumor periphery of PD gastric cancer patients, higher expression levels of SPP1, C1Q, and MIF are observed. **(D)** Quantitative analysis of fluorescence intensity for CD206, C1Q, SPP1, and MIF. Asterisks (*) indicate statistical significance, whereas “ns” denotes non-significance. **(E)** Tumor tissues from PD gastric cancer patients exhibit higher expression of immune checkpoint molecules PD1 and TIM3, as well as elevated expression of MIF. **(F)** Quantitative analysis of fluorescence intensity for CD208, PD1, TIM3, and MIF. Asterisks (*) indicate statistical significance, whereas “ns” denotes non-significance. **P < 0.01, and ***P < 0.001.

To verify the existence of a stromal-localized immunosuppressive network coordinated by Macro_SPP1/C1QC macrophages and CD8_Tex_C1 exhausted T cells in gastric cancer tissues, and to investigate the correlation between MIF expression and immunotherapy efficacy, we performed tissue immunofluorescence staining on gastric cancer biopsy specimens. In the tumor periphery of PD patients undergoing gastric cancer immunotherapy, higher expression levels of PD-1, SPP1, and C1Q were observed, while the abundance of CD8^+^ T cells showed no significant differences. This suggests that CD8^+^ T cells in the tumor periphery of PD patients predominantly exhibit an exhausted phenotype (**Figures 6A, B**). Additionally, CD206, a marker for M2-type macrophages, was also found to be highly expressed in PD patients (**Figures 6C, D**). Compared to PR patients, we further identified higher expression of the MIF molecule in PD patients, along with elevated levels of immune checkpoint molecules (PD-1 and TIM3) (**Figures 6E, F**).

### 3.6 Gastric cancer immune subtyping and precision therapy strategies exploring

**Figure 7:**
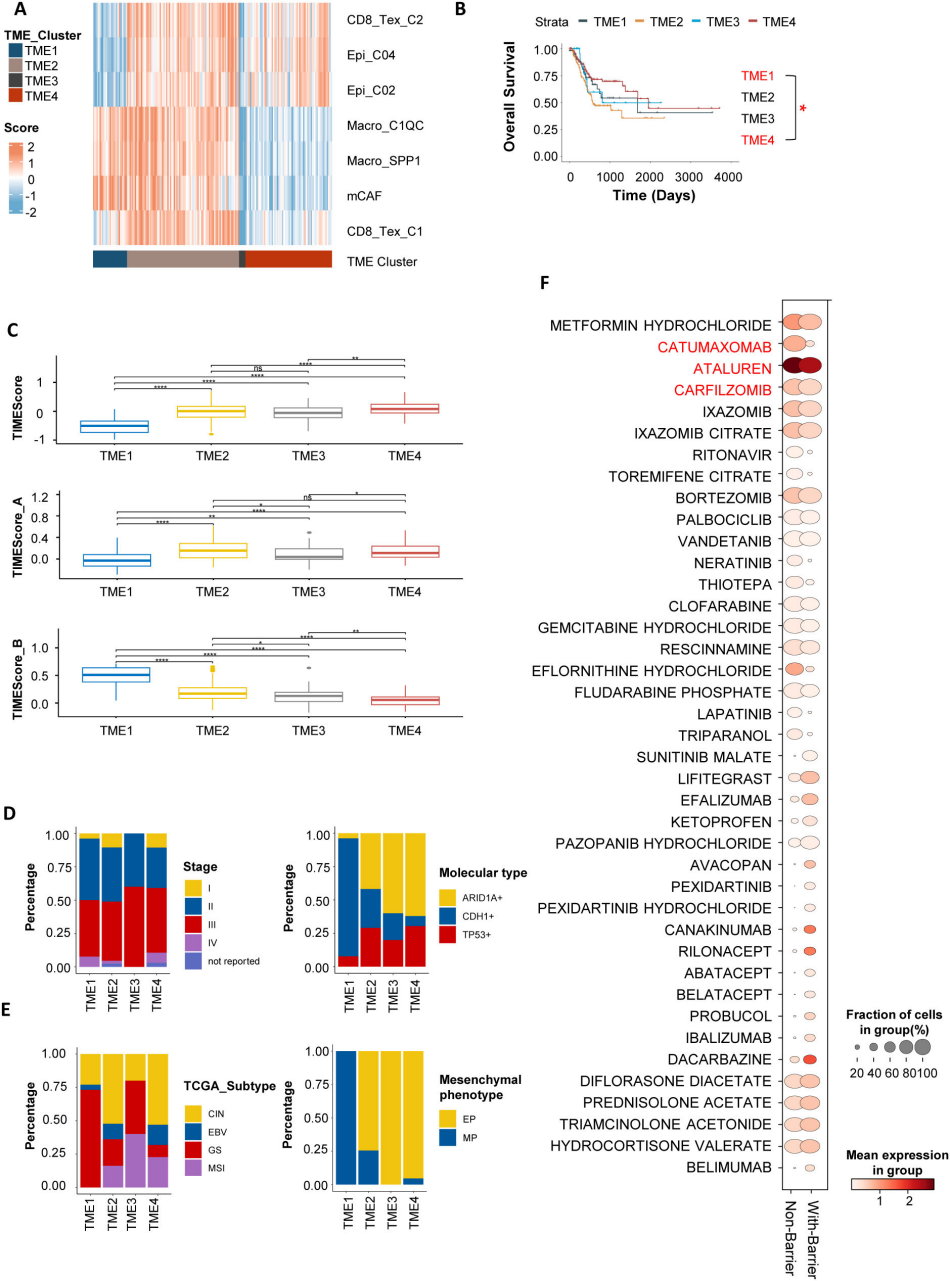
Gastric cancer patient subtyping based on barrier-associated features. **(A)** In TCGA-STAD, gastric cancer patients were subtyped into four tumor immune microenvironment (TME) clusters based on barrier-associated features. TME1 represents the “Immune Barrier Dominant” type; TME2, the “Immune Barrier with CD8^+^ T Cell Exhaustion” type; TME3, the “Immune Desert” type; and TME4, the “CD8^+^ T Cell Exhaustion Dominant” type. **(B)** Kaplan-Meier survival curves for the four TME subtypes. Asterisks (*) indicate statistical significance (*P* < 0.05), whereas “ns” denotes non-significance. Only TME1 and TME4 subtypes showed statistical significance in this analysis. **(C)** Box plots showing TMEScores for patients with different barrier-associated types. Higher TMEScores indicate a more favorable immune microenvironment and potential responsiveness to ICIs. **(D, E)** Bar charts displaying the distribution of the four barrier-associated types across different clinical stages and TCGA molecular subtypes. **(F)** Bubble plot summarizing potential targeted therapies for barrier-associated types cell types, predicted using the drug2cell tool. The Non-Barrier subtype involves cells such as CD8_Tex_C2, Epi_C04, and Epi_C02; the With-Barrier subtype involves cells including Macro_SPP1, Macro_C1QC, mCAF, and CD8_Tex_C2. A higher Mean expression in group indicates that the expression of genes in the cell marker genes that match these drug target genes is higher. A larger Fraction of cells in group suggests that the proportion of cell types targeted by a specific drug is greater. **P < 0.01, and ****P < 0.0001.

To explore the clinical significance and targeted therapeutic strategies of the immunosuppressive barrier, we evaluated the abundance of barrier-associated immune cells and their surrounding epithelial and stromal cells in the TCGA and ACRG gastric cancer transcriptome cohorts. We then performed unsupervised clustering to subtype patients based on immune profiles (**Figure 7A**; **Supplementary Figure S7A**, **Supplementary Tables S6, 7**). The results demonstrated that the gastric cancer microenvironment could be categorized into four distinct immune interaction patterns through analysis of the TCGA-STAD cohort: the “Immune Barrier Dominant” type (TME 1), the “Immune Barrier with CD8^+^ T Cell Exhaustion” type (TME 2), the “CD8^+^ T Cell Exhaustion Dominant” type (TME 4), and the “Immune Desert” type (TME 3). In the “Immunosuppressive Barrier Dominant” type, the microenvironment is primarily characterized by the existence of an immunosuppressive barrier, with limited CD8^+^ T cell infiltration. In contrast, the “Immunosuppressive Barrier with CD8^+^ T Cell Exhaustion” type features the presence of an immunosuppressive barrier along with some degree of CD8^+^ T cell Exhaustion.

Survival analysis revealed that patients with the “ Immunosuppressive Barrier with CD8^+^ T Cell Exhaustion “ and “Immunosuppressive Barrier Dominant” types had the worst prognosis, while those with the”CD8^+^ T Cell Exhaustion Dominant” had the best prognosis (**Figure 7B**; **Supplementary Figure S6B**). Further analysis of the TMEscore—a gastric cancer immune efficacy prediction score developed by our team—showed that patients with the “ CD8^+^ T Cell Exhaustion Dominant “ type had higher TMEscores, indicating better immune therapy responses, whereas those with the “Immunosuppressive Barrier with CD8^+^ T Cell Exhaustion “ had the lowest TMEscores, suggesting immune resistance (**Figure 7C**; **Supplementary Figure S6C**). In the TCGA dataset, clinical feature (34, 35) analysis showed that patients with genomically stable (GS) gastric cancer had the highest proportion of the “Immunosuppressive Barrier with CD8^+^ T Cell Exhaustion” type and were associated with poorer prognosis (**Figures 7D, E**). These results indicate that different stages or molecular subtypes of gastric cancer exhibit distinct “Immunosuppressive Barrier with CD8^+^ T Cell Exhaustion” subtypes in the tumor microenvironment, corresponding to differential therapeutic efficacy and survival outcomes.

Given the immunosuppressive barrier - associated characteristics of gastric cancer, the development of personalized immune combination therapy strategies is of vital importance. CD8_Tex_C1 cells mainly express PD - 1 as the immune checkpoint molecule, whereas CD8_Tex_C2 is distinguished by CTLA - 4 expression (**Supplementary Figure S5C**). To further explore this, we utilized the Drug2cell tool to classify the gastric cancer microenvironment into two groups based on the presence or absence of an immunosuppressive barrier. The barrier - associated group was marked by the presence of Macro_SPP1, Macro_C1QC, CD8_Tex_C1, and mCAFs cells. In contrast, the non - barrier group was characterized by CD8_Tex_C2, Epi_C02, and Epi_C04 cells. Subsequently, we predicted potential therapeutic drugs associated with the barrier-related subtypes (**Figure 7F**), which included agents in the anti-tumor (e.g., Catumaxomab, Carfilzomib), antiviral (e.g., Ritonavir, Ibalizumab), and immune-modulating (e.g., Abatacept, Belatacept) categories. Among these predicted drugs, some may have the potential to target both the Non-Barrier and With-Barrier subtypes, such as Ataluren. However, these drug predictions were made using the drug2cell tool, which matches cell gene signatures with drug target genes. The actual efficacy of these drugs requires further validation through *in vitro* and *in vivo* experiments.

## 4 Discussion

The tumor immune microenvironment (TIME), comprising diverse immune cells, has intricate interactions that significantly impact the progression and immune resistance of gastric cancer (36). (GC). In this study, we integrated single-cell sequencing, spatial transcriptomics, and bulk RNA sequencing data to conduct a comprehensive analysis of 20 immune cell subtypes. These subtypes were classified into three functional modules: anti-tumor (M1), pro-tumor (M2), and anti-promoting interaction (M3). Further analysis suggested the presence of an immunosuppressive barrier in gastric cancer, which may contribute to immune dysfunction. This barrier appears to involve interactions among Macro_SPP1, Macro_C1QC, and CD8_Tex_C1 cells within the stromal area.

Based on single-cell sequencing, this study elucidated the dynamic interactions of immune cells in the gastric cancer TIME. In the T/NK cell compartment, CD8_Tex_C1/C2 cells were enriched in patients with progressive disease (PD) and were associated with poor prognosis (33). In contrast, CD8_Teff cells maintained anti-tumor activity by secreting IFN-γ and TNF-α, showing a positive correlation with immune therapy response (37, 38). In the B cell compartment, B_Stress cells mediated immune evasion through the NR4A family and heat shock proteins, while Bregs shaped an immunosuppressive microenvironment via TGF-β (39). Among myeloid cells, Macro_C1QC and Macro_SPP1 (with M2-like features) dominated immune dysfunction and were closely related to T cell exhaustion and poor prognosis (40).

Analysis of the immune cell interaction network showed that the anti-tumor module (M1) was driven by cDC2 cells for antigen presentation, while the pro-tumor module (M2) consisted of a core motif formed by Macro_SPP1, Macro_C1QC, and CD8_Tex_C1 cells, which dominated immune dysfunction and were regulated by the interaction module (M3). This network structure recapitulated the tumor immune cycle of “antigen presentation-effector killing-terminal exhaustion” and revealed that TAMs-T cell interactions may be a key node in immune resistance, providing a new direction for TIME remodeling (41–43).

The Macro_C1QC and Macro_SPP1 cell subpopulations exhibited significant functional heterogeneity and collaboratively shaped an immunosuppressive microenvironment. Previous studies have shown that Macro_C1QC inhibits CD8^+^ T cell function through C1q complement signaling and lipid metabolism reprogramming mediated by FABP5 (44), while Macro_SPP1 exacerbates T cell exhaustion and promotes tumor metastasis via SPP1-CD44, hypoxia-HIF-1α axis, and MIF signaling (45, 46). Additionally, THBS2^+^ CAFs (Thrombospondin-2^+^ CAFs) can promote the conversion of Macro_C1QC to Macro_SPP1 through the C3/C3AR1 axis, indicating dynamic transformation potential between the two and shared dysregulation of cholesterol metabolism (Ch25h/25HC pathway) (47). This study further revealed that both Macro_C1QC and Macro_SPP1 highly expressed APOE and mediated immune therapy resistance through MIF-CD74/CXCR4 signaling. Moreover, similar to the TIB structure in liver cancer (46), these two subpopulations were found to form an immunosuppressive barrier in conjunction with cancer-associated fibroblasts (CAFs). This barrier not only physically impedes the infiltration of CD8^+^ T cells into the tumor, but also intensifies their exhaustion via immunosuppressive signaling. Consequently, this dual mechanism enhances tumor immune evasion and contributes to the development of resistance to immunotherapy in gastric cancer.

In addition, our study identified the differentiation trajectories of the CD8_Tex_C1/C2 subpopulations and their potential for immunotherapy. CD8_Tex_C1 cells partially retained cytotoxicity (GZMB/GZMK) and simultaneously expressed exhaustion-related genes such as PDCD1, acting as precursors to exhaustion (48). In contrast, CD8_Tex_C2 cells exhibited terminal exhaustion features, with attenuated effector functions and high expression of immune checkpoints. These two subpopulations were mediated by distinct transcriptional regulatory networks (Tex_C1: TEAD1/KLF13; Tex_C2: E2F family), and their spatial distribution showed an evolutionary trend from the tumor periphery stroma (Tex_C1) to the tumor core (Tex_C2), revealing the coupling between exhaustion and T cell migration (49). Based on these findings, we speculate that the CD8_Tex_C2 cells within the tumor may originate from the migration and transformation of CD8_Tex_C1 cells from the peripheral stroma, undergoing functional alterations in the process. Additionally, we found differences in the immune checkpoint profiles of the Tex_C1/C2 subpopulations (Tex_C1 enriched for VSIR/TNFRSF9, Tex_C2 enriched for TNFSF14/CTLA4), suggesting their specific exhaustion pathways and providing new strategies for targeting differentiation trajectories and reversing immune resistance.

In terms of the specific molecular mechanisms, we observed that in patients who did not respond to immunotherapy (NR group), the signaling network axes within the barrier-associated immune cells, which include MIF-CD74/CXCR4/CD44, LGALS9-CD45, HLA-CD8 and CXCL16-CXCR6, was significantly activated. Previous studies have shown that the MIF signaling axis shapes an immunosuppressive TIME through the ERK1/2, AMPK, and AKT pathways and enhances tumor tolerance to oxidative stress (50–52). MIF inhibitors (such as IPG1576) can reduce MDSC differentiation (53), while the LGALS9-CD45/SPP1-CD44 axis promotes Macro_C1QC infiltration and CD8^+^T cell exhaustion (45), and the CXCL16-CXCR6 axis drives T cell functional failure and gastric cancer metastasis (54). In the NFHGC cohort, HLA-CD8 signaling was upregulated in CD8_Tex_C1 cells within the NR group, and in CD8_Tex_C2 cells in both NR and R groups. This signaling axis represents a fundamental component of MHC-I antigen presentation and CD8^+^ T cell recognition. However, the functional relevance of its upregulation remains unclear. Given the presence of concurrent immunosuppressive signals such as MIF and LGALS9, it is possible that this interaction reflects persistent antigen exposure or dysfunctional immune engagement, rather than effective cytotoxic activation. Therefore, targeting these signaling pathways, such as inhibiting MIF isomerase activity or blocking the CXCL16-CXCR6 axis, may enhance immunotherapy responses and optimize the clinical efficacy of PD-1/PD-L1 combination therapies.

Finally, leveraging the barrier-associated features, we categorized patients into four distinct groups: the “Immune Barrier Dominant” type (characterized by immunosuppression and poor prognosis), the “CD8^+^ T Cell Exhaustion Dominant” type (marked by better immune infiltration and potential sensitivity to immune checkpoint blockade [ICB]), the “Immune Barrier with CD8^+^ T Cell Exhaustion” type, and the “Immune Desert” type. This classification system offers an alternative approach that may help to overcome some of the spatial resolution limitations encountered with the previously developed TMEScore tool by our team (10). TCGA molecular subtype analysis indicated that patients with the GS subtype had a higher proportion of those exhibiting an immunosuppressive barrier, which may explain their poor prognosis (55, 56), while the MP subtype corresponded to the “ Immune Barrier Dominant” type and the EP subtype was consistent with the “ CD8^+^ T Cell Exhaustion Dominant” type, suggesting the potential for chemotherapy combined with immunotherapy (57). EBV^+^/MSI^+^ patients were enriched in the “CD8^+^ T Cell Exhaustion Dominant” type, indicating that EBV^+^ patients could consider combination with LAG3 inhibitors, and MSI^+^ patients are suitable for PD-1 monotherapy (58–60). Additionally, for different T cell exhaustion subpopulations (CD8_Tex_C1/C2), tailored PD-1/LAG3 or CTLA-4/OX40 targeting strategies are needed. Metformin (61–65), which regulates metabolism, reverses exhaustion, and reshapes macrophage polarization, shows potential for cross-subtype intervention of the both barrier-associated and non-barrier gastric patients.

While this study has uncovered the role of the immunosuppressive barrier in immune resistance in gastric cancer, further *in vitro* and *in vivo* experiments are necessary to facilitate clinical translation. Additionally, the dynamic evolution of the immune system under therapeutic pressure, particularly the state transitions and proportion changes of immune cells, was not fully elucidated in this study. In this study, we applied a stringent quality control workflow—including cell filtering, mitochondrial gene thresholding, and batch correction algorithms—to reduce technical variation and enable robust integration of samples across patients. Nonetheless, residual batch effects and gene dropout remain intrinsic limitations of current single-cell technologies (66, 67). Moreover, the limited sample size of spatial transcriptomics and the absence of matched single-cell sequencing data before and after immunotherapy constrained the in-depth exploration of the underlying mechanisms.

## 5 Conclusion

Our study has identified a potential stromal immunosuppressive barrier in gastric cancer, characterized by the presence of Macro_SPP1/C1QC macrophages and CD8_Tex_C1 T cells. This barrier may contribute to immune dysfunction and therapy resistance. Molecular subtyping based on the presence of this barrier could inform personalized immune therapy strategies. Additionally, we found that macrophage-derived MIF signaling appears to drive immunosuppression via the MIF-CD74/CXCR4/CD44 axis.

## Data Availability

The original data are available from the corresponding authors upon reasonable request. Single-cell RNA sequencing (scRNA-Seq) and spatial transcriptomic data were obtained from the GEO database (accession numbers GSE183904 and GSE251950). Bulk RNA-Seq data for the melanoma ICB therapy cohort and the ACRG gastric tumor study were retrieved from GEO (accession numbers GSE100797 and GSE62254). The KIM cohort data were sourced from the TIDE website.

## Glossary

ACRG: Asian Cancer Research Group
CAFs: Cancer-Associated Fibroblast Cells
CD8_Teff: CD8^+^ Effector T Cells
CD8_Tex: CD8^+^ Exhausted T Cells
CTLs: Cytotoxic T Lymphocytes
DCs: Dendritic Cells
FS: Favorability Score
GEO: The Gene Expression Omnibus
GS: Genomically Stable
ICI: Immune Checkpoint Inhibitor
IC: Immune Checkpoint Blockade
GC: Gastric Cancer
Macro_C1QC: C1QC^+^ Macrophages
Macro_SPP1: SPP1^+^Macrophages
MIF: Macrophage Migration Inhibitory Factor
MDSCs: Myeloid-Derived Suppressor Cells
Mono_FCN1: FCN1^+^ Monocytes
NFHGC Cohort: Nanfang Hospital Gastric Cancer Cohort
NR: Non-Responders
OS: Overall Survival
ORR: Objective Response Rate
PC: Plasma Cells
R: Responders
STAD: Stomach Adenocarcinoma
ST: Spatial Transcriptomics
TIDE: Tumor Immune Dysfunction and Exclusion
TIME: Tumor Immune Microenvironment
TME: Tumor Microenvironment
TAMs: Tumor-Associated Macrophages
TaNK: Tumor-Associated NK Cells
TCR: T Cell Receptor
Tregs: Regulatory T Cells.

## References

[1] Joshi SS Badgwell BD. Current treatment and recent progress in gastric cancer. CA: Cancer J Clin. (2021) 71:264–79. doi: 10.3322/caac.21657, PMID: 33592120 PMC9927927

[2] Yasuda T Wang YA. Gastric cancer immunosuppressive microenvironment heterogeneity: implications for therapy development. Trends Cancer. (2024) 10:627–42. doi: 10.1016/j.trecan.2024.03.008, PMID: 38600020 PMC11292672

[3] Luo D Zhou J Ruan S Zhang B Zhu H Que Y. Overcoming immunotherapy resistance in gastric cancer: insights into mechanisms and emerging strategies. Cell Death Dis. (2025) 16:75. doi: 10.1038/s41419-025-07385-7, PMID: 39915459 PMC11803115

[4] Huang KK Ma H Chong RHH Uchihara T Lian BSX Zhu F. Spatiotemporal genomic profiling of intestinal metaplasia reveals clonal dynamics of gastric cancer progression. Cancer Cell. (2023) 41:2019–37.e8. doi: 10.1016/j.ccell.2023.10.004, PMID: 37890493 PMC10729843

[5] Luo Q Dong Z Xie W Fu X Lin L Zeng Q. Apatinib remodels the immunosuppressive tumor ecosystem of gastric cancer enhancing anti-pd-1 immunotherapy. Cell Rep. (2023) 42:112437. doi: 10.1016/j.celrep.2023.112437, PMID: 37097818

[6] Tsutsumi C Ohuchida K Katayama N Yamada Y Nakamura S Okuda S. Tumor-infiltrating monocytic myeloid-derived suppressor cells contribute to the development of an immunosuppressive tumor microenvironment in gastric cancer. Gastric cancer: Off J Int Gastric Cancer Assoc Japanese Gastric Cancer Assoc. (2024) 27:248–62. doi: 10.1007/s10120-023-01456-4, PMID: 38217732

[7] Lee SY Jhun J Woo JS Lee KH Hwang SH Moon J. Gut microbiome-derived butyrate inhibits the immunosuppressive factors pd-L1 and il-10 in tumor-associated macrophages in gastric cancer. Gut Microbes. (2024) 16:2300846. doi: 10.1080/19490976.2023.2300846, PMID: 38197259 PMC10793689

[8] Qu Y Wang X Bai S Niu L Zhao G Yao Y. The effects of tnf-α/tnfr2 in regulatory T cells on the microenvironment and progression of gastric cancer. Int J Cancer. (2022) 150:1373–91. doi: 10.1002/ijc.33873, PMID: 34766338 PMC9298834

[9] Li Y Zheng Y Huang J Nie RC Wu QN Zuo Z. Caf-macrophage crosstalk in tumour microenvironments governs the response to immune checkpoint blockade in gastric cancer peritoneal metastases. Gut. (2025) 74:350–63. doi: 10.1136/gutjnl-2024-333617, PMID: 39537239 PMC11874311

[10] Zeng D Wu J Luo H Li Y Xiao J Peng J. Tumor microenvironment evaluation promotes precise checkpoint immunotherapy of advanced gastric cancer. J immunotherapy Cancer. (2021) 9. doi: 10.1136/jitc-2021-002467, PMID: 34376552 PMC8356190

[11] Zeng D Li M Zhou R Zhang J Sun H Shi M. Tumor microenvironment characterization in gastric cancer identifies prognostic and immunotherapeutically relevant gene signatures. Cancer Immunol Res. (2019) 7:737–50. doi: 10.1158/2326-6066.Cir-18-0436, PMID: 30842092

[12] Kim ST Cristescu R Bass AJ Kim KM Odegaard JI Kim K. Comprehensive molecular characterization of clinical responses to pd-1 inhibition in metastatic gastric cancer. Nat Med. (2018) 24:1449–58. doi: 10.1038/s41591-018-0101-z, PMID: 30013197

[13] Shi M Zeng D Luo H Xiao J Li Y Yuan X. Tumor microenvironment rna test to predict immunotherapy outcomes in advanced gastric cancer: the times001 trial. Med (New York NY). (2024) 5:1378–92.e3. doi: 10.1016/j.medj.2024.07.006, PMID: 39089261

[14] Sun Y Chen S Lu Y Xu Z Fu W Yan W. Single-cell transcriptomic analyses of tumor microenvironment and molecular reprograming landscape of metastatic laryngeal squamous cell carcinoma. Commun Biol. (2024) 7:63. doi: 10.1038/s42003-024-05765-x, PMID: 38191598 PMC10774275

[15] Qin P Chen H Wang Y Huang L Huang K Xiao G. Cancer-associated fibroblasts undergoing neoadjuvant chemotherapy suppress rectal cancer revealed by single-cell and spatial transcriptomics. Cell Rep Med. (2023) 4:101231. doi: 10.1016/j.xcrm.2023.101231, PMID: 37852187 PMC10591051

[16] Chen C Guo Q Liu Y Hou Q Liao M Guo Y. Single-cell and spatial transcriptomics reveal postn(+) cancer-associated fibroblasts correlated with immune suppression and tumour progression in non-small cell lung cancer. Clin Trans Med. (2023) 13:e1515. doi: 10.1002/ctm2.1515, PMID: 38115703 PMC10731139

[17] Cable DM Murray E Zou LS Goeva A Macosko EZ Chen F. Robust decomposition of cell type mixtures in spatial transcriptomics. Nat Biotechnol. (2022) 40:517–26. doi: 10.1038/s41587-021-00830-w, PMID: 33603203 PMC8606190

[18] Wang R Song S Qin J Yoshimura K Peng F Chu Y. Evolution of immune and stromal cell states and ecotypes during gastric adenocarcinoma progression. Cancer Cell. (2023) 41:1407–26.e9. doi: 10.1016/j.ccell.2023.06.005, PMID: 37419119 PMC10528152

[19] Zheng L Qin S Si W Wang A Xing B Gao R. Pan-cancer single-cell landscape of tumor-infiltrating T cells. Sci (New York NY). (2021) 374:abe6474. doi: 10.1126/science.abe6474, PMID: 34914499

[20] Fitzsimons E Qian D Enica A Thakkar K Augustine M Gamble S. A pan-cancer single-cell rna-seq atlas of intratumoral B cells. Cancer Cell. (2024) 42:1784–97.e4. doi: 10.1016/j.ccell.2024.09.011, PMID: 39406187

[21] Badia IMP Vélez Santiago J Braunger J Geiss C Dimitrov D Müller-Dott S. Decoupler: ensemble of computational methods to infer biological activities from omics data. Bioinf Adv. (2022) 2:vbac016. doi: 10.1093/bioadv/vbac016, PMID: 36699385 PMC9710656

[22] Kanemaru K Cranley J Muraro D Miranda AMA Ho SY Wilbrey-Clark A. Spatially resolved multiomics of human cardiac niches. Nature. (2023) 619:801–10. doi: 10.1038/s41586-023-06311-1, PMID: 37438528 PMC10371870

[23] Jiang C Chen J Sun T Xu J Zhu H Chen J. Unveiling dynamic changes and regulatory mechanisms of T cell subsets in sepsis pathogenesis. ImmunoTargets Ther. (2024) 13:29–44. doi: 10.2147/itt.S448691, PMID: 38322277 PMC10844014

[24] Mayer S Milo T Isaacson A Halperin C Miyara S Stein Y. The tumor microenvironment shows a hierarchy of cell-cell interactions dominated by fibroblasts. Nat Commun. (2023) 14:5810. doi: 10.1038/s41467-023-41518-w, PMID: 37726308 PMC10509226

[25] Li C Zhang B Schaafsma E Reuben A Wang L Turk MJ. Timigp: inferring cell-cell interactions and prognostic associations in the tumor immune microenvironment through gene pairs. Cell Rep Med. (2023) 4:101121. doi: 10.1016/j.xcrm.2023.101121, PMID: 37467716 PMC10394258

[26] Lee SH Lee D Choi J Oh HJ Ham IH Ryu D. Spatial dissection of tumour microenvironments in gastric cancers reveals the immunosuppressive crosstalk between ccl2+ Fibroblasts and stat3-activated macrophages. Gut. (2024) 74(5):714–27. doi: 10.1136/gutjnl-2024-332901, PMID: 39580151 PMC12013559

[27] Zhang M Hu S Min M Ni Y Lu Z Sun X. Dissecting transcriptional heterogeneity in primary gastric adenocarcinoma by single cell rna sequencing. Gut. (2021) 70:464–75. doi: 10.1136/gutjnl-2019-320368, PMID: 32532891 PMC7873416

[28] Kumar V Ramnarayanan K Sundar R Padmanabhan N Srivastava S Koiwa M. Single-cell atlas of lineage states, tumor microenvironment, and subtype-specific expression programs in gastric cancer. Cancer Discov. (2022) 12:670–91. doi: 10.1158/2159-8290.Cd-21-0683, PMID: 34642171 PMC9394383

[29] Sun S Li JY Nim HT Piers A Ramialison M Porrello ER. Cd90 marks a mesenchymal program in human thymic epithelial cells *in vitro* and *in vivo* . Front Immunol. (2022) 13:846281. doi: 10.3389/fimmu.2022.846281, PMID: 35371075 PMC8966383

[30] True LD Zhang H Ye M Huang CY Nelson PS von Haller PD. Cd90/thy1 is overexpressed in prostate cancer-associated fibroblasts and could serve as a cancer biomarker. Modern pathology: an Off J United States Can Acad Pathology Inc. (2010) 23:1346–56. doi: 10.1038/modpathol.2010.122, PMID: 20562849 PMC2948633

[31] Khoo TK Coenen MJ Schiefer AR Kumar S Bahn RS. Evidence for enhanced thy-1 (Cd90) expression in orbital fibroblasts of patients with graves’ Ophthalmopathy. Thyroid: Off J Am Thyroid Assoc. (2008) 18:1291–6. doi: 10.1089/thy.2008.0255, PMID: 18976167 PMC2857447

[32] Khaliq AM Rajamohan M Saeed O Mansouri K Adil A Zhang C. Spatial transcriptomic analysis of primary and metastatic pancreatic cancers highlights tumor microenvironmental heterogeneity. Nat Genet. (2024) 56:2455–65. doi: 10.1038/s41588-024-01914-4, PMID: 39294496

[33] Beltra JC Manne S Abdel-Hakeem MS Kurachi M Giles JR Chen Z. Developmental relationships of four exhausted cd8(+) T cell subsets reveals underlying transcriptional and epigenetic landscape control mechanisms. Immunity. (2020) 52:825–41.e8. doi: 10.1016/j.immuni.2020.04.014, PMID: 32396847 PMC8360766

[34] Li X Wu WK Xing R Wong SH Liu Y Fang X. Distinct subtypes of gastric cancer defined by molecular characterization include novel mutational signatures with prognostic capability. Cancer Res. (2016) 76:1724–32. doi: 10.1158/0008-5472.Can-15-2443, PMID: 26857262

[35] Li B Zhang F Niu Q Liu J Yu Y Wang P. A molecular classification of gastric cancer associated with distinct clinical outcomes and validated by an xgboost-based prediction model. Mol Ther Nucleic Acids. (2023) 31:224–40. doi: 10.1016/j.omtn.2022.12.014, PMID: 36700042 PMC9843270

[36] Zavros Y Merchant JL. The immune microenvironment in gastric adenocarcinoma. Nat Rev Gastroenterol Hepatol. (2022) 19:451–67. doi: 10.1038/s41575-022-00591-0, PMID: 35288702 PMC9809534

[37] Huang H Huang Z Ge J Yang J Chen J Xu B. Cd226 identifies functional cd8(+)T cells in the tumor microenvironment and predicts a better outcome for human gastric cancer. Front Immunol. (2023) 14:1150803. doi: 10.3389/fimmu.2023.1150803, PMID: 37056782 PMC10086426

[38] Guo X Nie H Zhang W Li J Ge J Xie B. Contrasting cytotoxic and regulatory T cell responses underlying distinct clinical outcomes to anti-pd-1 plus lenvatinib therapy in cancer. Cancer Cell. (2025) 43:248–68.e9. doi: 10.1016/j.ccell.2025.01.001, PMID: 39889705

[39] Jansen K Cevhertas L Ma S Satitsuksanoa P Akdis M van de Veen W. Regulatory B cells, a to Z. Allergy. (2021) 76:2699–715. doi: 10.1111/all.14763, PMID: 33544905

[40] Toledo B Zhu Chen L Paniagua-Sancho M Marchal JA Perán M Giovannetti E. Deciphering the performance of macrophages in tumour microenvironment: A call for precision immunotherapy. J Hematol Oncol. (2024) 17:44. doi: 10.1186/s13045-024-01559-0, PMID: 38863020 PMC11167803

[41] Waibl Polania J Hoyt-Miggelbrink A Tomaszewski WH Wachsmuth LP Lorrey SJ Wilkinson DS. Antigen presentation by tumor-associated macrophages drives T cells from a progenitor exhaustion state to terminal exhaustion. Immunity. (2025) 58:232–46.e6. doi: 10.1016/j.immuni.2024.11.026, PMID: 39724910

[42] van Weverwijk A de Visser KE. Mechanisms driving the immunoregulatory function of cancer cells. Nat Rev Cancer. (2023) 23:193–215. doi: 10.1038/s41568-022-00544-4, PMID: 36717668

[43] Chu Y Dai E Li Y Han G Pei G Ingram DR. Pan-cancer T cell atlas links a cellular stress response state to immunotherapy resistance. Nat Med. (2023) 29:1550–62. doi: 10.1038/s41591-023-02371-y, PMID: 37248301 PMC11421770

[44] Zhang S Peng W Wang H Xiang X Ye L Wei X. C1q(+) tumor-associated macrophages contribute to immunosuppression through fatty acid metabolic reprogramming in Malignant pleural effusion. J immunotherapy Cancer. (2023) 11. doi: 10.1136/jitc-2023-007441, PMID: 37604643 PMC10445384

[45] Peng H Jiang L Yuan J Wu X Chen N Liu D. Single-cell characterization of differentiation trajectories and drug resistance features in gastric cancer with peritoneal metastasis. Clin Trans Med. (2024) 14:e70054. doi: 10.1002/ctm2.70054, PMID: 39422697 PMC11488346

[46] Liu Y Xun Z Ma K Liang S Li X Zhou S. Identification of a tumour immune barrier in the hcc microenvironment that determines the efficacy of immunotherapy. J Hepatol. (2023) 78:770–82. doi: 10.1016/j.jhep.2023.01.011, PMID: 36708811

[47] Xiao J Wang S Chen L Ding X Dang Y Han M. 25-hydroxycholesterol regulates lysosome amp kinase activation and metabolic reprogramming to educate immunosuppressive macrophages. Immunity. (2024) 57:1087–104.e7. doi: 10.1016/j.immuni.2024.03.021, PMID: 38640930

[48] Sathe A Grimes SM Lau BT Chen J Suarez C Huang RJ. Single-cell genomic characterization reveals the cellular reprogramming of the gastric tumor microenvironment. Clin Cancer research: an Off J Am Assoc Cancer Res. (2020) 26:2640–53. doi: 10.1158/1078-0432.Ccr-19-3231, PMID: 32060101 PMC7269843

[49] Lan X Zebley CC Youngblood B. Cellular and molecular waypoints along the path of T cell exhaustion. Sci Immunol. (2023) 8:eadg3868. doi: 10.1126/sciimmunol.adg3868, PMID: 37656775 PMC10618911

[50] Jankauskas SS Wong DWL Bucala R Djudjaj S Boor P. Evolving complexity of mif signaling. Cell signalling. (2019) 57:76–88. doi: 10.1016/j.cellsig.2019.01.006, PMID: 30682543

[51] Li Y Wang Z Lu F Miao Y Feng Q Zhu W. Novel T cell exhaustion gene signature to predict prognosis and immunotherapy response in thyroid carcinoma from integrated rna-sequencing analysis. Sci Rep. (2024) 14:8375. doi: 10.1038/s41598-024-58419-7, PMID: 38600248 PMC11006682

[52] Chen J Guo W Du P Cui T Yang Y Wang Y. Mif inhibition alleviates vitiligo progression by suppressing cd8(+) T cell activation and proliferation. J Pathol. (2023) 260:84–96. doi: 10.1002/path.6073, PMID: 36852981

[53] Jia X Xi J Tian B Zhang Y Wang Z Wang F. The tautomerase activity of tumor exosomal mif promotes pancreatic cancer progression by modulating mdsc differentiation. Cancer Immunol Res. (2024) 12:72–90. doi: 10.1158/2326-6066.Cir-23-0205, PMID: 37956411

[54] Han J Fu R Chen C Cheng X Guo T Huangfu L. Cxcl16 promotes gastric cancer tumorigenesis via adam10-dependent cxcl16/cxcr6 axis and activates akt and mapk signaling pathways. Int J Biol Sci. (2021) 17:2841–52. doi: 10.7150/ijbs.57826, PMID: 34345211 PMC8326113

[55] Cancer Genome Atlas Research Network. Comprehensive molecular characterization of gastric adenocarcinoma. Nature. (2014) 513:202–9. doi: 10.1038/nature13480, PMID: 25079317 PMC4170219

[56] Cristescu R Lee J Nebozhyn M Kim KM Ting JC Wong SS. Molecular analysis of gastric cancer identifies subtypes associated with distinct clinical outcomes. Nat Med. (2015) 21:449–56. doi: 10.1038/nm.3850, PMID: 25894828

[57] Oh SC Sohn BH Cheong JH Kim SB Lee JE Park KC. Clinical and genomic landscape of gastric cancer with a mesenchymal phenotype. Nat Commun. (2018) 9:1777. doi: 10.1038/s41467-018-04179-8, PMID: 29725014 PMC5934392

[58] Suh YS Na D Lee JS Chae J Kim E Jang G. Comprehensive molecular characterization of adenocarcinoma of the gastroesophageal junction between esophageal and gastric adenocarcinomas. Ann Surg. (2022) 275:706–17. doi: 10.1097/sla.0000000000004303, PMID: 33086305

[59] Saito M Kono K. Landscape of ebv-positive gastric cancer. Gastric cancer: Off J Int Gastric Cancer Assoc Japanese Gastric Cancer Assoc. (2021) 24:983–9. doi: 10.1007/s10120-021-01215-3, PMID: 34292431

[60] Muro K Chung HC Shankaran V Geva R Catenacci D Gupta S. Pembrolizumab for patients with pd-L1-positive advanced gastric cancer (Keynote-012): A multicentre, open-label, phase 1b trial. Lancet Oncol. (2016) 17:717–26. doi: 10.1016/s1470-2045(16)00175-3, PMID: 27157491

[61] Wang S Lin Y Xiong X Wang L Guo Y Chen Y. Low-dose metformin reprograms the tumor immune microenvironment in human esophageal cancer: results of a phase ii clinical trial. Clin Cancer research: an Off J Am Assoc Cancer Res. (2020) 26:4921–32. doi: 10.1158/1078-0432.Ccr-20-0113, PMID: 32646922

[62] Pernicova I Korbonits M. Metformin–mode of action and clinical implications for diabetes and cancer. Nat Rev Endocrinol. (2014) 10:143–56. doi: 10.1038/nrendo.2013.256, PMID: 24393785

[63] Huang X Sun T Wang J Hong X Chen H Yan T. Metformin reprograms tryptophan metabolism to stimulate cd8+ T-cell function in colorectal cancer. Cancer Res. (2023) 83:2358–71. doi: 10.1158/0008-5472.Can-22-3042, PMID: 37195082

[64] Valaee S Yaghoobi MM Shamsara M. Metformin inhibits gastric cancer cells metastatic traits through suppression of epithelial-mesenchymal transition in a glucose-independent manner. PloS One. (2017) 12:e0174486. doi: 10.1371/journal.pone.0174486, PMID: 28334027 PMC5363973

[65] Zheng Y Tian X Wang T Xia X Cao F Tian J. Long noncoding rna pvt1 regulates the immunosuppression activity of granulocytic myeloid-derived suppressor cells in tumor-bearing mice. Mol Cancer. (2019) 18:61. doi: 10.1186/s12943-019-0978-2, PMID: 30925926 PMC6441229

[66] Lan T Hutvagner G Lan Q Liu T Li J. Sequencing dropout-and-batch effect normalization for single-cell mrna profiles: A survey and comparative analysis. Briefings Bioinf. (2021) 22. doi: 10.1093/bib/bbaa248, PMID: 33073843

[67] Qiu P. Embracing the dropouts in single-cell rna-seq analysis. Nat Commun. (2020) 11:1169. doi: 10.1038/s41467-020-14976-9, PMID: 32127540 PMC7054558

