# Supplementary material for: Identification of a stromal immunosuppressive barrier orchestrated by SPP1+/C1QC+ macrophages and CD8+ exhausted T cells driving gastric cancer immunotherapy resistance
Source: Front Immunol. 2025 Jul 16;16:1618591. doi: 10.3389/fimmu.2025.1618591 (PMC12307293; doi:10.3389/fimmu.2025.1618591)
Supplement: Supplementary file 1 [file Table1.docx]

**Supplementary Figures:**

**Fig. S1 Identification of Immune Cells in Gastric Cancer**
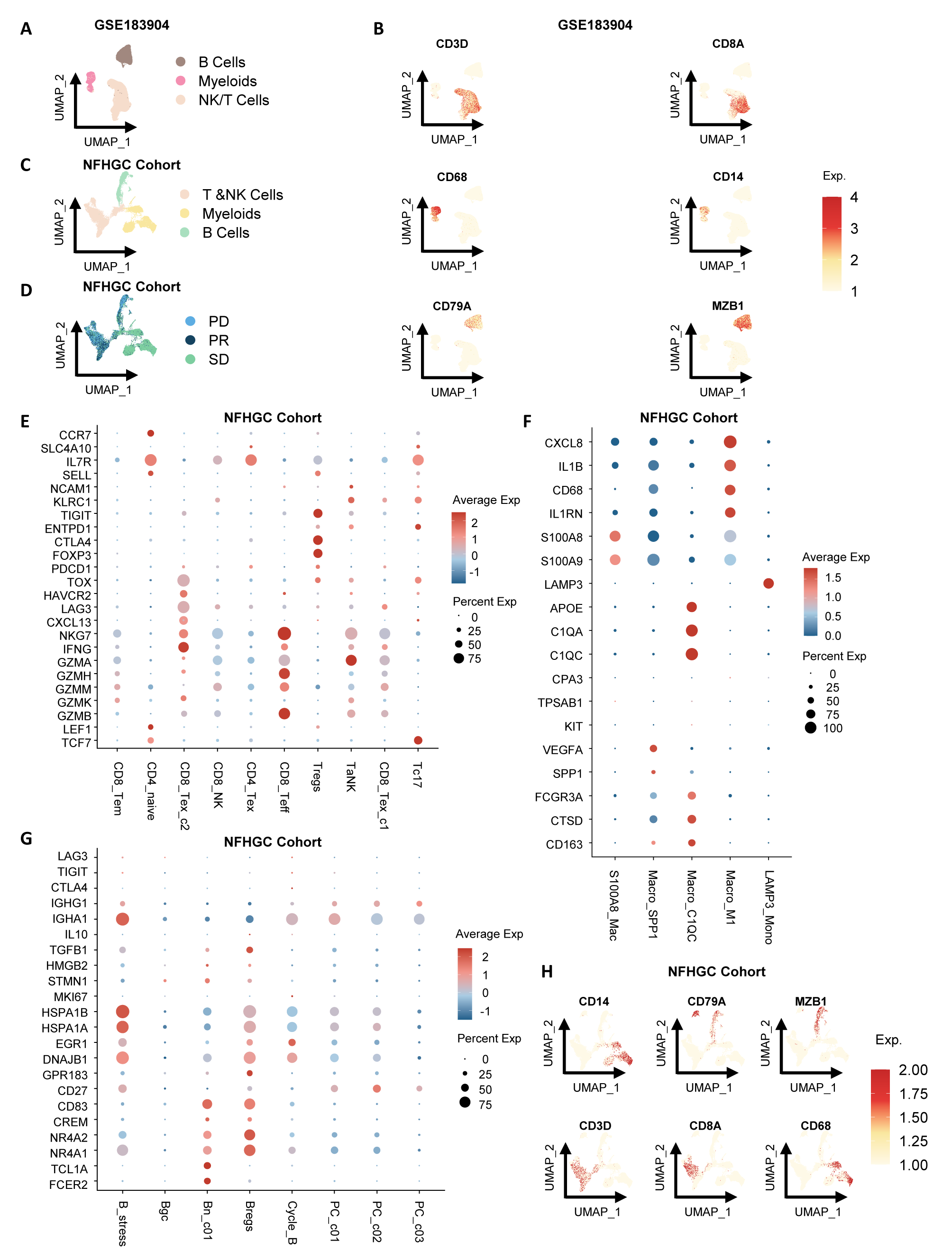


**A** The UMAP plot delineates the three major immune cell types identified in the GSE183904 dataset. **B** The UMAP plot highlights principal marker genes of immune cells identified in the GSE183904 dataset. **C** The UMAP plot delineates the three major immune cell types identified in the NFHGC Cohort. **D** The UMAP plot characterizes immune cell subclusters from patients with distinct therapeutic outcomes (PD, SD, PR) in the NFHGC cohort. PD indicates immunotherapy-responsive; SD and PR indicate non-responsive. **E–G** The dot plots display marker genes of immune cells identified in the NFHGC Cohort. **H** The UMAP plot highlights principal marker genes of immune cells identified in the NFHGC Cohort.

**Fig. S2 Immune Cell Communication Strength and Its Association with ICIs Efficacy in Melanoma Cohort**

**
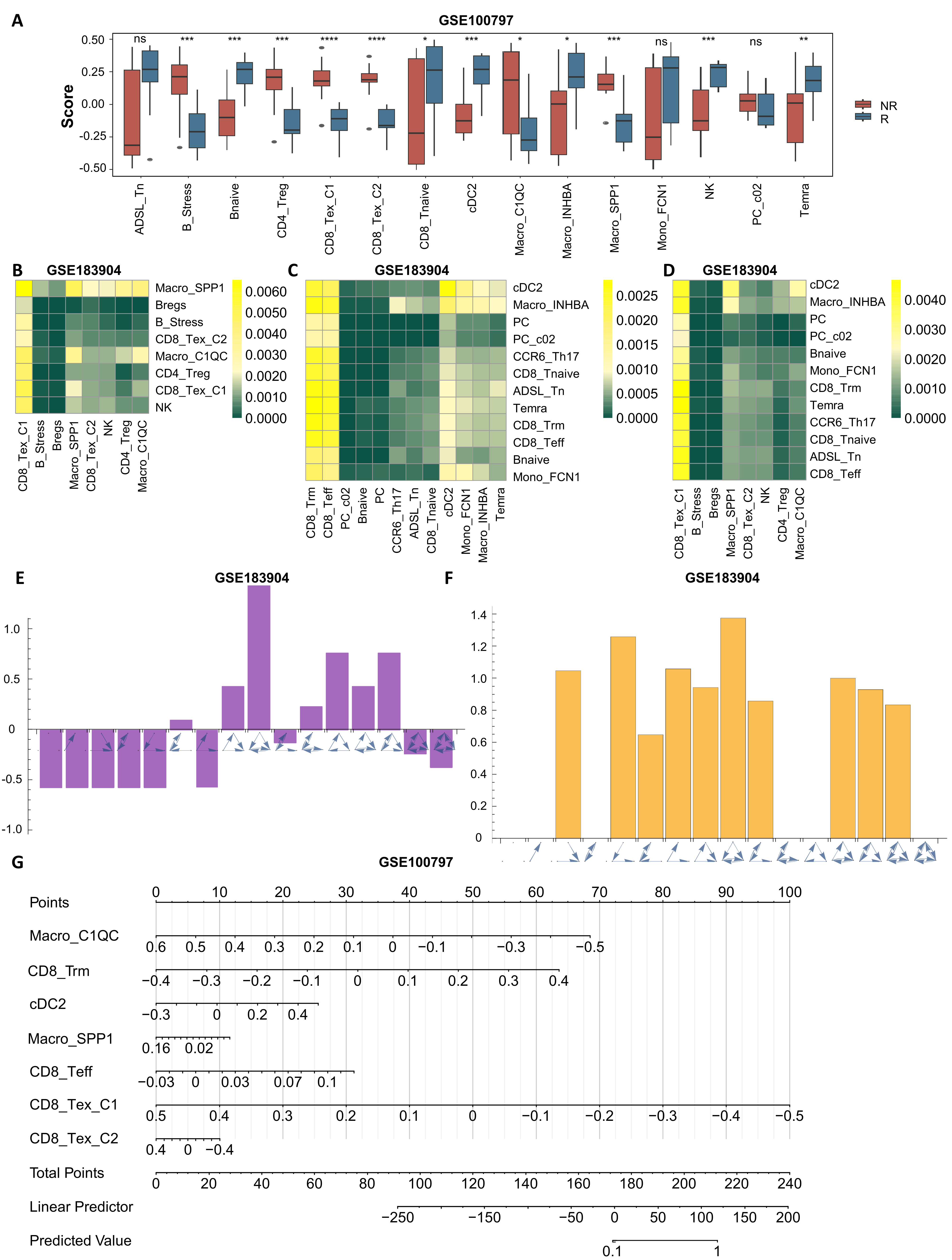
**

**A** Box plots showing differences in immune cell deconvolution scores between immunotherapy responders (R) and non-responders (NR) in the GSE100797 melanoma immunotherapy cohort. Asterisks (*) indicate statistical significance, whereas “ns” denotes non-significance. **B–D** Heatmaps depicting the strength of intercellular communication among immune cells, based on the CellChat algorithm. **B** Pro-tumoral immune cell modules(M1); **C** Anti-tumoral immune cell modules (M2); **D** Coordinated immune cell modules(M3). **E–F** Network motif analysis. Arrows on the x-axis represent different cell motif combinations. Higher motif scores indicate key cell interaction motifs within the network. **E** Pro-tumoral immune cell modules; **F** Anti-tumoral immune cell modules. **G** Nomogram illustrating the predictive ability of immune cells for immunotherapy response in the GSE100797 cohort. Higher Predicted Values indicate better immunotherapy response.

**Fig. S3 Immune Cell Abundance in Different Spatial Architectures**

**
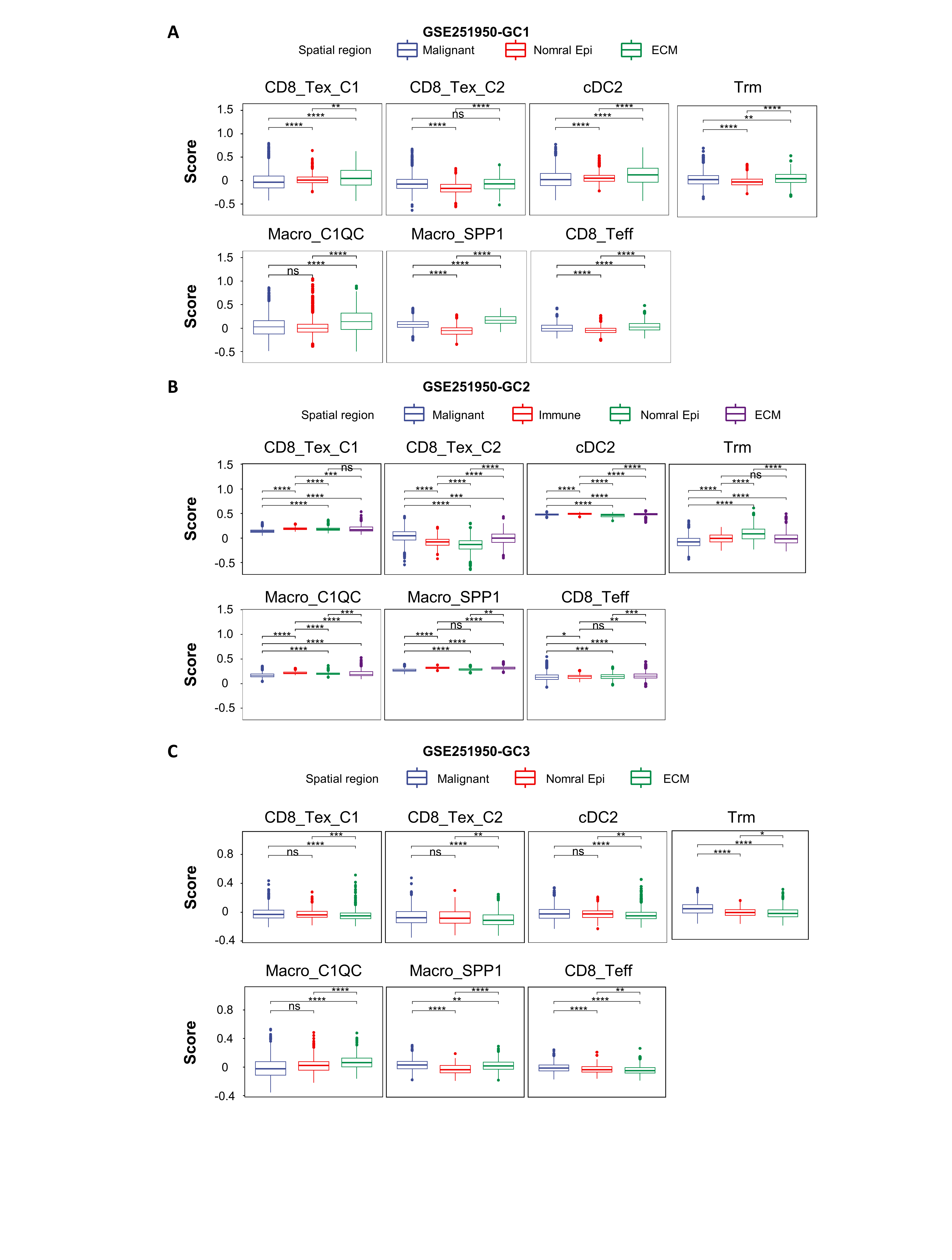
**

**A** Box plots showing immune cell abundance in malignant epithelial, normal epithelial, and extracellular matrix regions of the GC1 section. **B** Box plots showing immune cell abundance in malignant epithelial, immune, normal epithelial, and extracellular matrix regions of the GC2 section. **C** Box plots showing immune cell abundance in malignant epithelial, normal epithelial, and extracellular matrix regions of the GC3 section. Asterisks (*) indicate statistical significance, whereas “ns” denotes non-significance.

**Fig. S4 Identification and Spatial Co-localization Analysis of Epithelial and Stromal Cells**

**
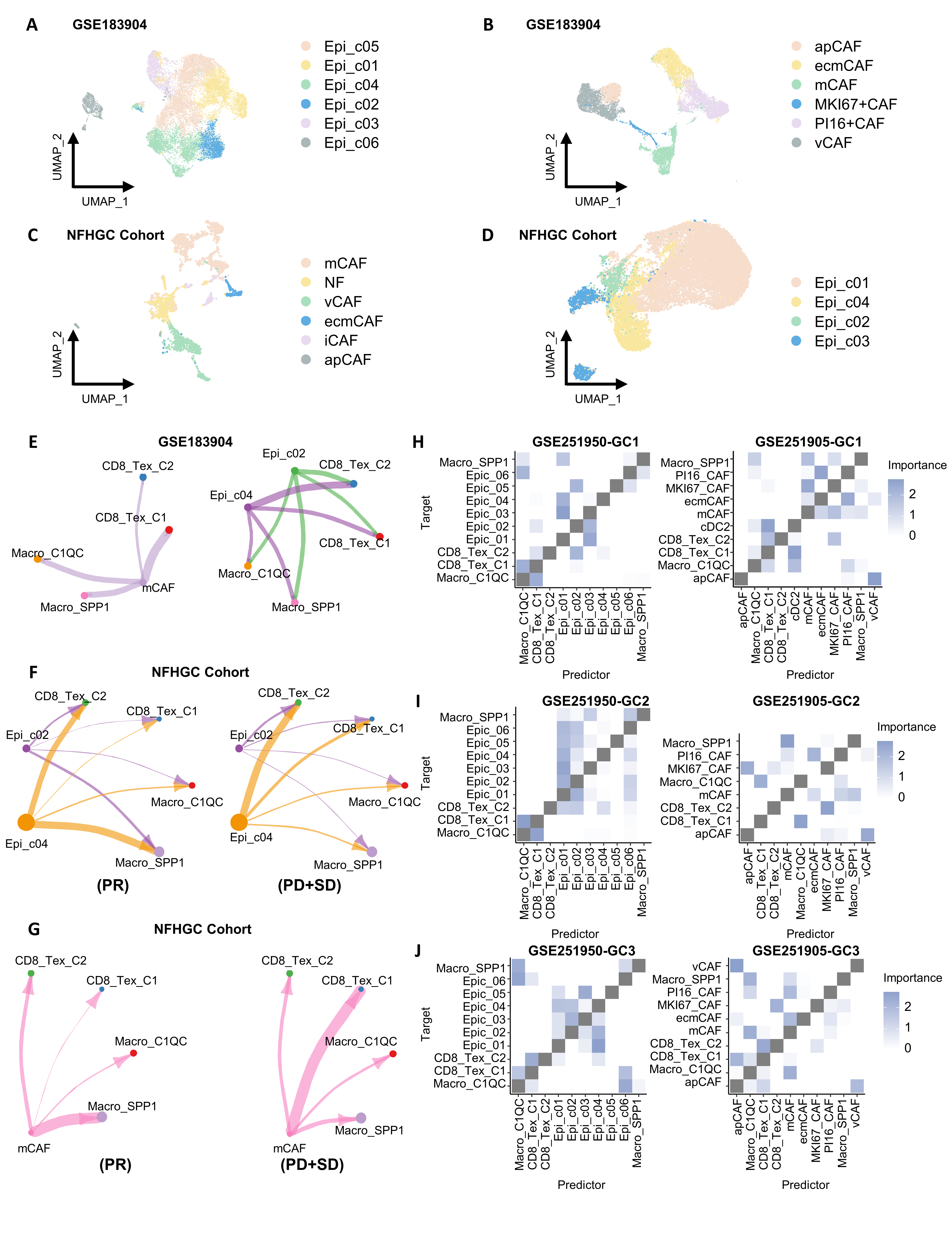
**

**A–B** UMAP plots depicting gastric cancer epithelial and stromal cell subclusters identified in the GSE183904 single-cell dataset. **C–D** UMAP plots depicting gastric cancer epithelial and stromal cell subclusters identified in the NFHGC Cohort. **E** Analysis of communication strength between mCAF subclusters and immune cells (CD8_Tex_C1, CD8_Tex_C2, Macro_SPP1, Macro_C1QC) in the GSE183904 single-cell dataset, as well as between Epi_C02 and Epi_C04 subclusters and the aforementioned immune cells. **F–G** Analysis of communication strength between mCAF subclusters and immune cells (CD8_Tex_C1, CD8_Tex_C2, Macro_SPP1, Macro_C1QC) in the NFHGC Cohort, stratified by immunotherapy response. **F** mCAF subclusters; **G** Epi_C02 and Epi_C04 subclusters. **H–J** Heatmaps showing the spatial co-localization importance scores calculated by the Misty algorithm in spatial transcriptomic data. Higher importance scores indicate stronger spatial co-localization between predictor and target cells. **H** Results for GC1 sample; **I** Results for GC2 sample; **J** Results for GC3 sample.

**Fig. S5 Differential Analysis of CD8+ T Cells in the GSE183904 Dataset**

**
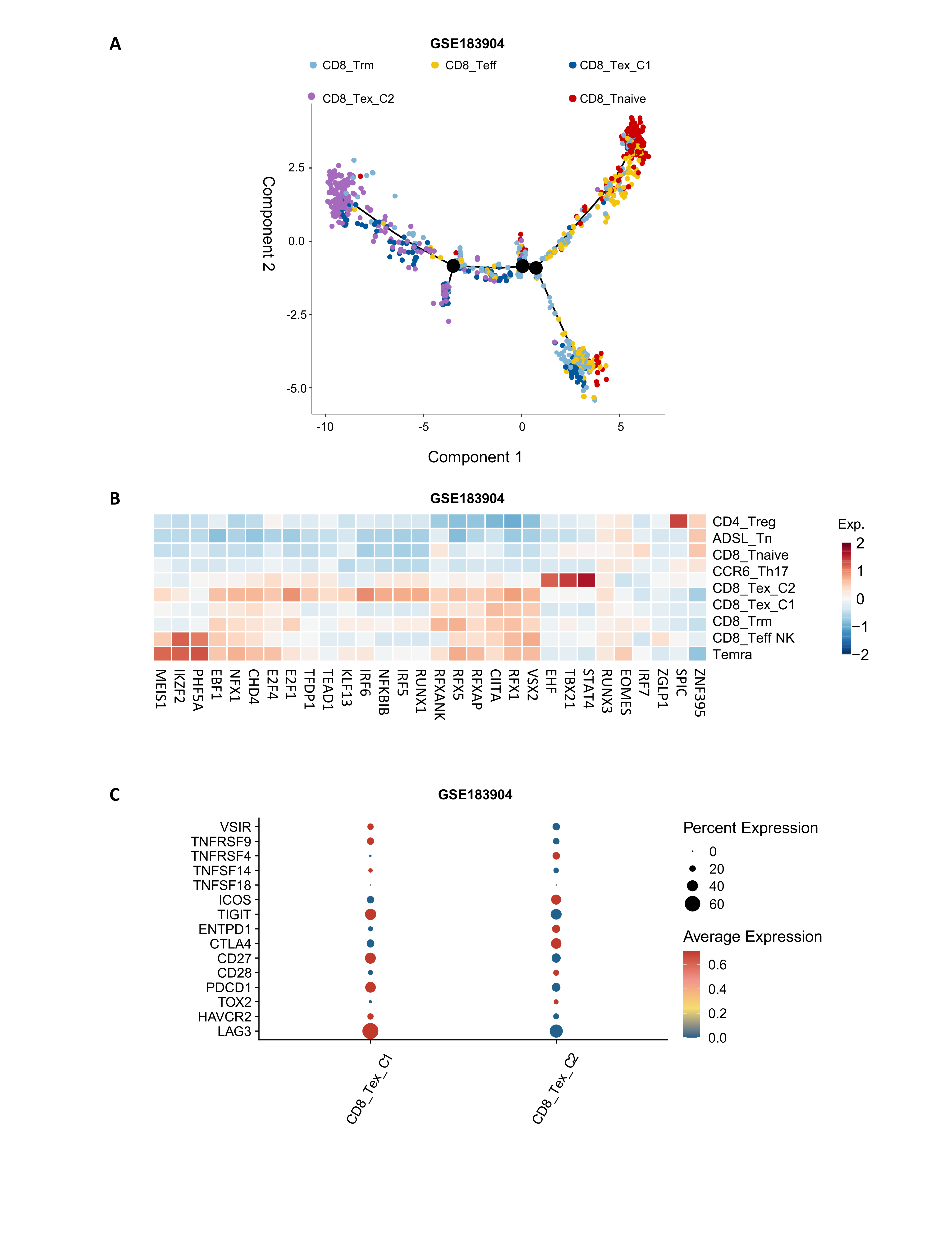
**

**A** Trajectory analysis of CD8^+^T cell subclusters. **B** Gene heatmap displaying transcription factors associated with distinct CD8^+^T cell subclusters. **C** Dot plot highlighting differences in immune checkpoint expression between CD8_Tex_C1 and CD8_Tex_C2 subclusters.

**Fig. S6 Gastric Cancer Patient Subtyping Based on Barrier-associated Features in ACRG Cohort
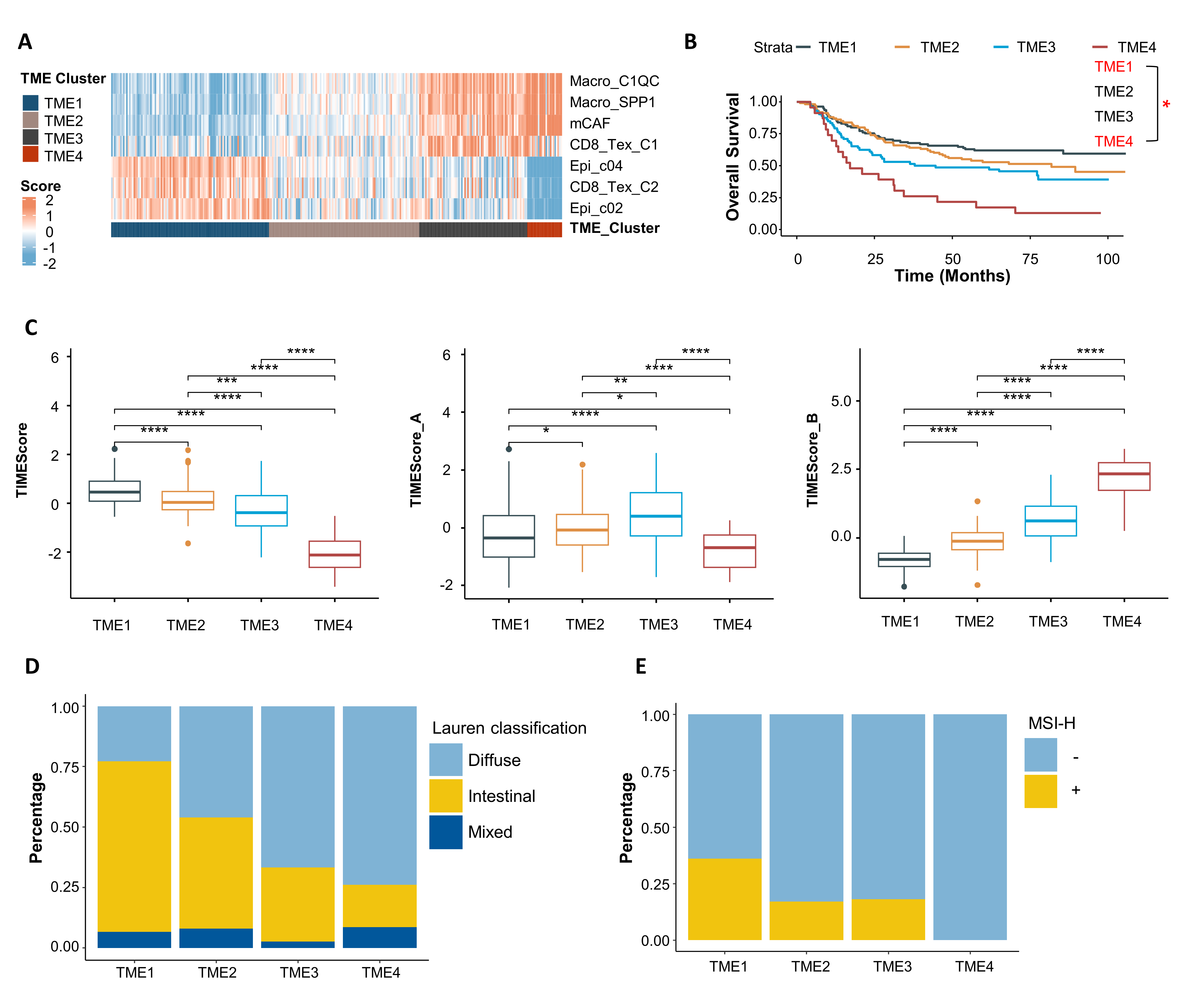
**

**A** In the ACRG Cohort, gastric cancer patients were subtyped into four tumor immune microenvironment (TME) clusters based on barrier-associated features. TME1 represents the “CD8^+^ T Cell Exhaustion Dominant” type; TME2, the “Immune Desert” type; TME3, the“Immune Barrier with CD8^+^ T Cell Exhaustion” type; and TME4, the “Immune Barrier Dominant” type. **B** Kaplan-Meier survival curves for the four barrier-associated subtypes. **C** Box plots showing TMEScores for patients with different barrier-associated types. Higher TMEScores indicate a more favorable immune microenvironment and potential responsiveness to ICIs. **D–E** Bar charts displaying the distribution of the barrier-associated types across different gastric molecular subtypes in the ACRG cohort.

**Fig. S7 Display of Marker Genes for Cellular Subpopulations in the GSE183904 Dataset and NFHGC Cohort**


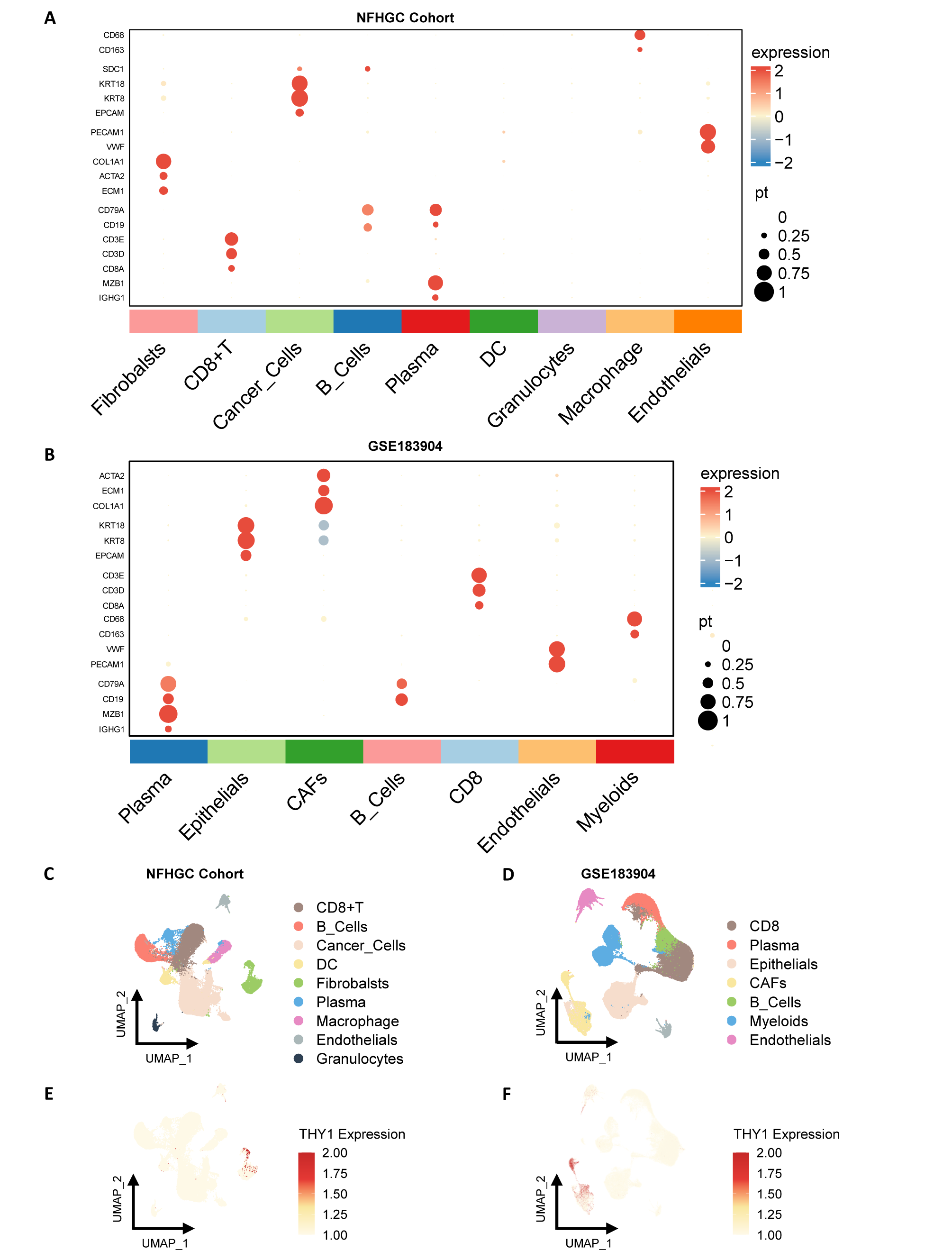


**A** Heatmap showing marker genes for single-cell subpopulations in the NFHGC cohort. **B** Heatmap showing marker genes for single-cell subpopulations in the GSE183904 dataset. **C-D** UMAP plots displaying single-cell subpopulations in the NFHGC cohort and GSE183904 dataset. **E–F** UMAP plots showing the expression of the *THY1* gene within single-cell subpopulations in the NFHGC cohort and GSE183904 dataset.
